# ANKRD22 is an N-myristoylated hairpin-like monotopic membrane protein specifically localized to lipid droplets

**DOI:** 10.1038/s41598-021-98486-8

**Published:** 2021-09-28

**Authors:** Toshihiko Utsumi, Takuro Hosokawa, Mayu Shichita, Misato Nishiue, Natsuko Iwamoto, Haruna Harada, Aya Kiwado, Manami Yano, Motoaki Otsuka, Koko Moriya

**Affiliations:** 1 Graduate School of Sciences and Technology for Innovation, Yamaguchi University, Yamaguchi, 753-8515 Japan; 2 Department of Biological Chemistry, Faculty of Agriculture, Yamaguchi University, Yamaguchi, 753-8515 Japan

**Keywords:** Biochemistry, Cancer, Cell biology, Molecular biology

## Abstract

The membrane topology and intracellular localization of ANKRD22, a novel human N-myristoylated protein with a predicted single-pass transmembrane domain that was recently reported to be overexpressed in cancer, were examined. Immunofluorescence staining of COS-1 cells transfected with cDNA encoding ANKRD22 coupled with organelle markers revealed that ANKRD22 localized specifically to lipid droplets (LD). Analysis of the intracellular localization of ANKRD22 mutants C-terminally fused to glycosylatable tumor necrosis factor (GLCTNF) and assessment of their susceptibility to protein N-glycosylation revealed that ANKRD22 is synthesized on the endoplasmic reticulum (ER) membrane as an N-myristoylated hairpin-like monotopic membrane protein with the amino- and carboxyl termini facing the cytoplasm and then sorted to LD. Pro98 located at the center of the predicted membrane domain was found to be essential for the formation of the hairpin-like monotopic topology of ANKRD22. Moreover, the hairpin-like monotopic topology, and positively charged residues located near the C-terminus were demonstrated to be required for the sorting of ANKRD22 from ER to LD. Protein N-myristoylation was found to positively affect the LD localization. Thus, multiple factors, including hairpin-like monotopic membrane topology, C-terminal positively charged residues, and protein N-myristoylation cooperatively affected the intracellular targeting of ANKRD22 to LD.

## Introduction

Protein N-myristoylation is one of the essential lipid modifications of protein found in eukaryotic and viral proteins. In this modification, a 14-carbon saturated fatty acid, myristic acid, is bound to the N-terminal Gly of protein after removal of the initiating Met^1–4^. Generally, protein N-myristoylation is a cotranslational irreversible modification of protein. In addition to the cotranslational protein N-myristoylation, posttranslational N-myristoylation was found to occur on many caspase-cleavage products in apoptotic cells^5–7^. The N-myristoyltransferase (NMT) catalyzes both cotranslational and posttranslational N-myristoylation^8^. Many N-myristoylated proteins play essential roles in the regulation of a wide variety of signal transduction pathways in cells. Besides these cellular signal transduction proteins, protein N-myristoylation is found on many disease-associated proteins^9–11^. In many cases, the reversible membrane binding mediated by protein N-myristoylation regulates the functions of these proteins. Thus, protein N-myristoylation is regarded as a lipid modification occurring mainly on cytoplasmic soluble proteins, but few integral membrane proteins have been found to be N-myristoylated.

In our previous studies, we established a strategy to comprehensively identify the human N*-*myristoylated proteins from human cDNA clones in human cDNA resources. In this strategy, N-myristoylated proteins were identified by metabolic labeling and mass spectrometric analyses of proteins obtained by an in vitro translation system^12,13^. Using this strategy, we identified ~ 30 novel N-myristoylated proteins out of approximately 6300 human cDNA clones^12–14^. Among these N-myristoylated proteins, we found two integral membrane proteins, SAMM50 and protein Lunapark (Lunapark). SAMM50 is a β-barrel protein localized in the outer membrane of mitochondria, and is a major component of the SAM complex required for the sorting and assembly of β-barrel proteins^15^. We investigated the role of protein N-myristoylation in the intracellular localization of SAMM50 and found that protein N-myristoylation is indispensable for the targeting of SAMM50 to mitochondria^16^. Thus, protein N-myristoylation can mediate targeting of mammalian integral transmembrane protein to mitochondria. Lunapark is an N-myristoylated double-pass integral membrane protein of the ER that is involved in ER network formation^17^. Based on our studies, protein N-myristoylation does not affect the membrane integration, membrane topology formation, or localization to ER of Lunapark. However, protein N-myristoylation was found to play essential roles in ER morphological changes induced by Lunapark^17^. Thus, in addition to mediating mitochondrial targeting, protein N-myristoylation plays an important role in the specific physiological functions of N-myristoylated membrane proteins. Considering these experimental results, protein N-myristoylation may mediate diverse roles in the intracellular localization and function of N-myristoylated integral membrane proteins.

In the present study, we further searched for novel human N-myristoylated transmembrane proteins from human proteins listed in the UniProtKB/Swiss-Prot protein database using a strategy established in our previous studies^12–14^. As a result, ANKRD22, a functionally unknown protein with a predicted single-pass transmembrane domain that was recently reported to be overexpressed in cancer, was found to be an N-myristoylated hairpin-like monotopic membrane protein specifically localized to lipid droplets (LD). Furthermore, multiple factors, including hairpin-like monotopic membrane topology, C-terminal positively charged residues, and protein N-myristoylation cooperatively affected the intracellular targeting of ANKRD22 to LD.

## Results

### ANKRD22, a functionally unknown putative single-pass transmembrane protein, is N-myristoylated

**Figure 1:**
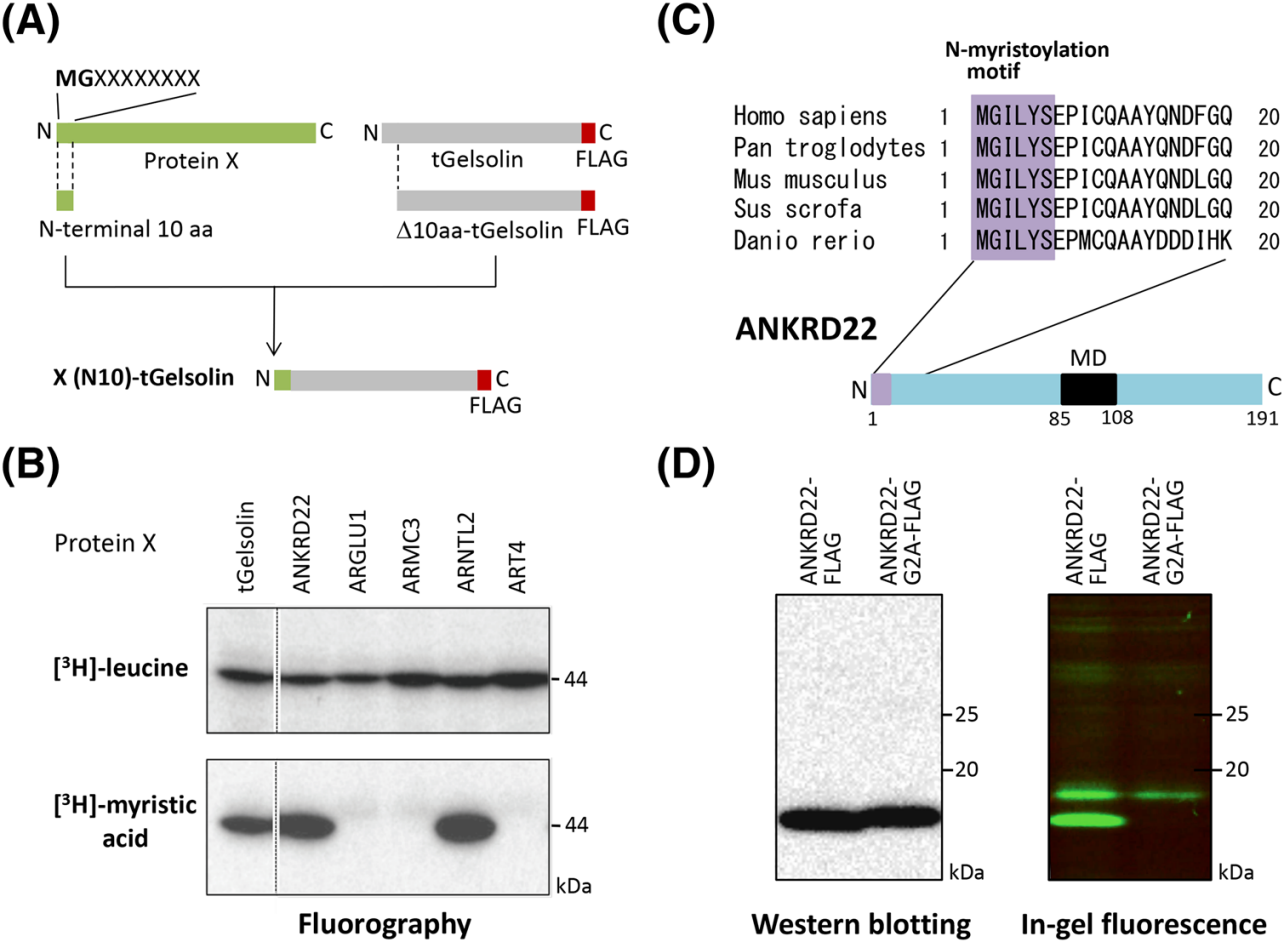
ANKRD22, a functionally unknown putative single-pass membrane protein, is N-myristoylated. Five candidate proteins (ANKRD22, ARGLU1, ARMC3, ARNTL2, and ART4) for novel human N-myristoylated proteins were selected from proteins listed in the UniProtKB/Swiss-Prot protein database, and their susceptibility to protein N-myristoylation was evaluated by in vitro and in vivo metabolic labeling. (**A**) Schematic representation of the generation of X(N10)-tGelsolin-FLAG with 10 N-terminal amino acids of the candidate protein X at its N-terminus. (**B**) Detection of protein N-myristoylation of X(N10)-tGelsolin-FLAGs by metabolic labeling in an insect cell-free protein synthesis system. X(N10)-tGelsolin-FLAGs were synthesized in the presence of [^3^H]leucine or [^3^H]myristic acid, and the labeled translation products were analyzed by SDS-PAGE and fluorography, as described in the “Methods”. A part of the fluorogram was cropped. The positions of the splices are indicated by vertical dashed lines. The uncropped raw image data for the fluorogram are shown in Supplementary Figure S7. (**C**) Structure of ANKRD22. Interspecies alignment of the N-terminal sequences of ANKRD22 is shown. N-myristoylation motifs are shown in purple in the N-terminal sequences. The predicted transmembrane domain is indicated as a black box. (**D**) Detection of protein N-myristoylation of ANKRD22 by metabolic labeling in transfected COS-1 cells. cDNA encoding ANKRD22-FLAG and ANKRD22-G2A-FLAG was transfected into COS-1 cells. The expression of proteins was evaluated by Western blotting using an anti-FLAG antibody. Protein N-myristoylation was evaluated by metabolic labeling with a myristic acid analog followed by click chemistry, as described in the “Methods”. The blot obtained by Western blotting was cropped. The raw image data for the full-length blot is shown in Supplementary Figure S7.

We first searched for novel human N-myristoylated transmembrane proteins from human proteins listed in the UniProtKB/Swiss-Prot protein database using a strategy established in our previous studies^12–14^. By this strategy, ~ 3700 proteins with N-terminal Met-Gly motifs were extracted from ~ 46,000 human proteins listed in the UniProtKB/Swiss-Prot protein database. After applying the N-terminal sequences of these proteins to two protein N-myristoylation prediction programs, the MYR Predictor and Myristoylator^18,19^, ~ 670 positively predicted proteins were identified. From these positively predicted proteins, candidate proteins for novel N-myristoylated proteins were selected and their susceptibility to protein N-myristoylation was evaluated using fusion proteins in which the 10 N-terminal amino acid residues were fused to a FLAG-tagged model protein, tGelsolin, as previously described^12^ (Fig. 1A). In this experiment, the fusion proteins were synthesized using an insect cell-free protein synthesis system in the presence of [^3^H]leucine or [^3^H]myristic acid, and then analyzed by SDS-PAGE and fluorography. The results of 5 candidate proteins (ANKRD22, ARGLU1, ARMC3, ARNTL2, and ART4) are shown in Fig. 1B. As shown in the upper panel, all proteins, including tGelsolin, a control N-myristoylated protein, were expressed based on the incorporation of [^3^H]leucine. In contrast, efficient incorporation of [^3^H]myristic acid comparable to that of control N-myristoylated tGelsolin was observed in selective fusion proteins such as ANKRD22(N10)-tGelsolin and ARNTL2(N10)-tGelsolin. Bioinformatic analysis of these proteins using SOSUI, a prediction system for membrane proteins^20^, suggested that ANKRD22 is a single-pass integral membrane protein. ANKRD22 is a functionally unknown protein that was recently reported to be overexpressed in cancer such as non-small cell lung, colorectal, and breast cancer^21–23^. Therefore, in this study, protein N-myristoylation, membrane localization, intracellular localization, and membrane topology of ANKRD22 expressed in mammalian cells were examined.

As shown in Fig. 1C, the high conservation of the N-myristoylation motif was observed among vertebrates by the interspecies alignment of the N-terminal sequence of the protein. To determine whether ANKRD22 is N-myristoylated, cellular metabolic labeling experiments in COS-1 cells using Alk-Myr, a bioorthogonal myristic acid analog, were performed. In this experiment, after labeling cells transfected with cDNA encoding C-terminally FLAG-tagged ANKRD22 or its non-myristoylatable G2A mutant, with Alk-Myr, the N-myristoylated protein was detected with click chemistry. As shown in the left panel of Fig. 1D, efficient expression of protein in COS-1 cells was equally observed for the wild-type and G2A mutant forms of ANKRD22-FLAG according to Western blotting analysis. Metabolic labeling experiments revealed the efficient Alk-Myr incorporation in wild-type ANKRD22-FLAG, but such incorporation was abolished in G2A mutant (Fig. 1D, right panel). Thus, ANKRD22-FLAG expressed in COS-1 cells was N-myristoylated.

### ANKRD22 specifically localized to LD

**Figure 2:**
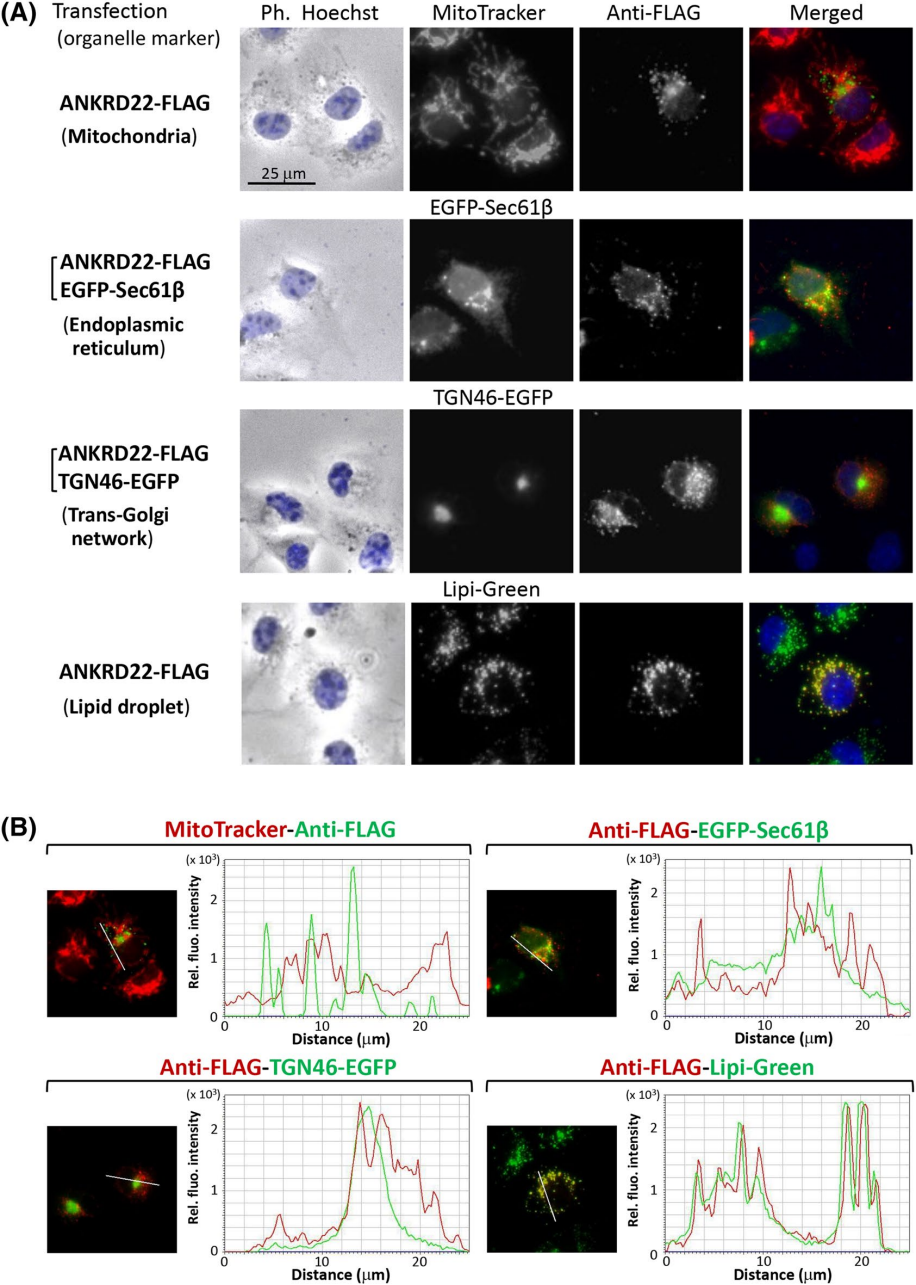
Analysis of the intracellular localization of ANKRD22. Intracellular localization of ANKRD22 was assessed by immunofluorescence analysis of COS-1 cells transfected with cDNA coding ANKRD22-FLAG using anti-FLAG antibody. Hoechst, MitoTracker Red, EGFP-Sec61β, TGN46-EGFP, and Lipi-Green were used as organelle markers for nucleus, mitochondria, ER, trans-Golgi network, and LD, respectively. The experiments were repeated 3 times and the similar results were obtained. A representative data was presented. (**A**) Phase contrast and fluorescence images obtained by immunofluorescence analysis. (**B**) The results of the line profile analysis of the fluorescence images obtained in (**A**). For this analysis, corresponding line-scan graphs of the relative fluorescence intensities of green fluorescence (green line) and red fluorescence (red line) along the white line indicated in the merged images are shown.

**Figure 3:**
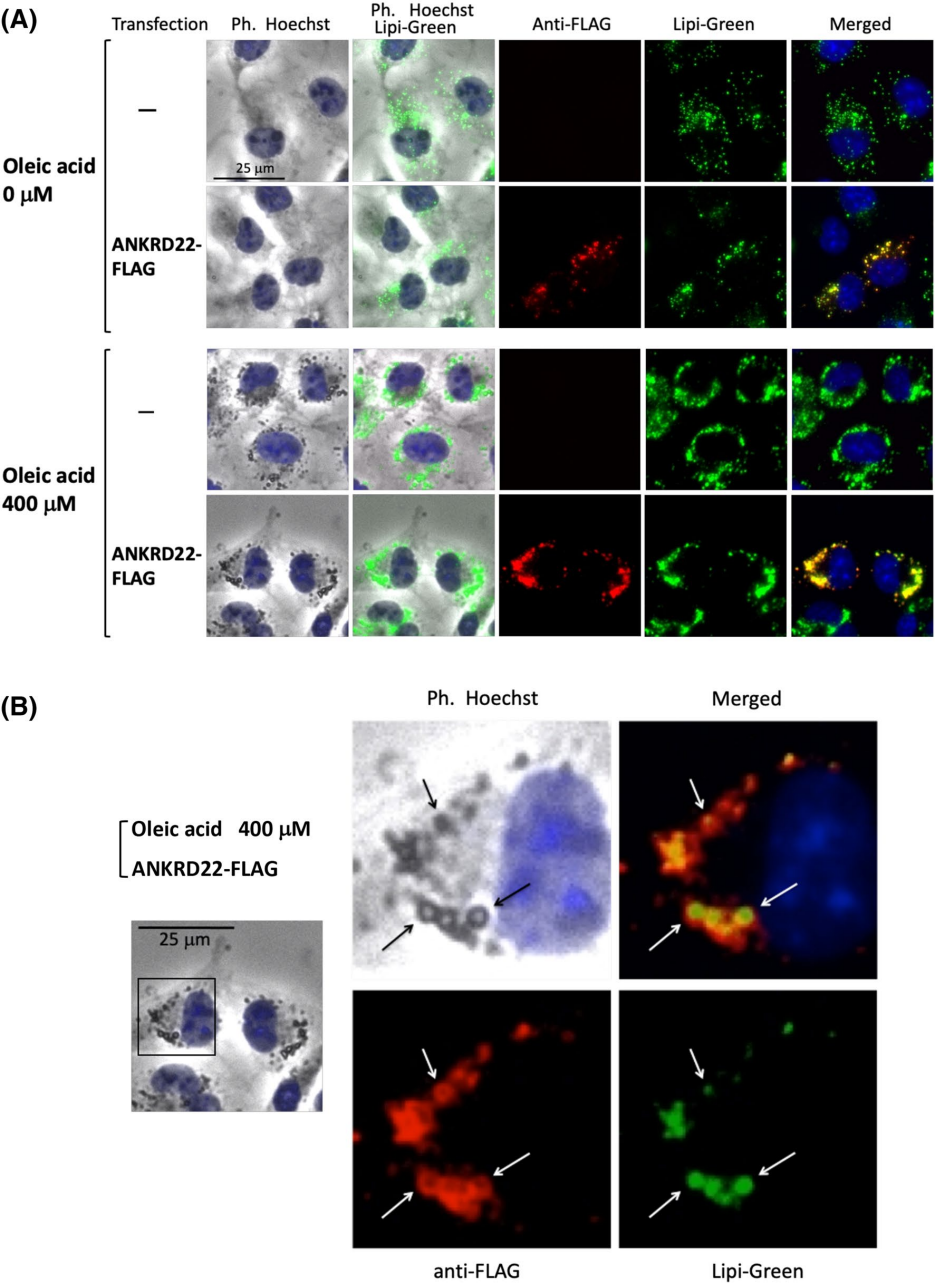
ANKRD22 specifically localized to LD. To confirm the specific localization of ANKRD22 to LD, LD formation in non-transfected COS-1 cells or COS-1 cells transfected with ANKRD22-FLAG cDNA was induced by incubating cells with a medium containing 400 µM oleic acid (OA) overnight, and the cells were then stained with Lipi-Green. Thereafter, the intracellular localization of ANKRD22-FLAG was assessed by immunofluorescence analysis. For this analysis, non-transfected COS-1 cells or transfected COS-1 cells incubated overnight in the absence of OA were used as control cells. The experiments were repeated 3 times and the similar results were obtained. A representative data was presented. (**A**) Phase contrast and fluorescence images obtained by immunofluorescence analysis of control (non-OA-treated) COS-1 cells and COS-1 cells treated with 400 µM OA. (**B**) Close-up images of phase contrast and fluorescence images of COS-1 cells transfected with ANKRD22-FLAG cDNA treated with 400 µM oleic acid (OA) shown in (**A**). Close-up images of the area surrounded by a black box in the left panel were shown.

In order to assess the intracellular localization of ANKRD22, immunofluorescence analysis of COS-1 cells transfected with cDNA coding ANKRD22-FLAG was performed. For this analysis, MitoTracker Red, EGFP-Sec61β, TGN46-EGFP, and Lipi-Green were used as the organelle markers for mitochondria, ER, trans-Golgi network, and LD, respectively. As shown in Fig. 2A, based on immunofluorescence analysis, overexpressed ANKRD22-FLAG exhibited a characteristic distribution pattern with dot-like structures in the cytoplasm that merged with Lipi-Green, a LD-specific fluorescent probe, but not with organelle markers for mitochondria, ER, or the trans-Golgi network^24^. This suggested that ANKRD22 specifically localized to LD. The line profile analysis of the obtained fluorescence images further confirmed the specific localization of ANKRD22-FLAG to LD as shown in Fig. 2B. When Nile Red, a well-known fluorescent marker for LD, was used as LD marker, similar results were obtained as shown in Supplementary Figure S1. In addition, the same experiment using human HepG2 cells (hepatocellular carcinoma cells) revealed that ANKRD22-FLAG expressed in human cells specifically localized to LD as shown in Supplementary Figure S2. When intracellular localization of endogenous ANKRD22 expressed in COS-1 cells was assessed by the immunofluorescence analysis using anti-ANKRD22 antibody, no obvious fluorescence signal derived from anti-ANKRD22 antibody was observed, suggesting that ANKRD22 was not efficiently expressed in COS-1 cells (Supplementary Figure S3, top panels). To confirm the LD localization of ANKRD22-FLAG, LD formation in COS-1 cells was induced by incubation with a medium containing 400 µM oleic acid (OA) overnight and then staining with Lipi-Green. As shown in the lower panels in Fig. 3A, newly formed LD were observed as black dots in phase contrast images of cells, which were efficiently stained with Lipi-Green. These black dots were not observed in control non-OA-treated COS-1 cells (Fig. 3A). When a similar experiment was performed with cells transfected with ANKRD22-FLAG cDNA, the expressed proteins were observed specifically in LD in the COS-1 cells (Fig. 3A, lower panels), demonstrating that ANKRD22 specifically localized to LD. When close-up images of a part of the image presented in Fig. 3A bottom panels was generated, ring-like structure was observed with immunofluorescence image using anti-FLAG antibody as indicated by white arrows in Fig. 3B, lower left panel. In this case, ring-like structure was also observed with phase contrast image of the same position (upper left panel). Furthermore, the ring-like structure was not observed with Lipi-Green staining of the same position (lower right panel). These results clearly indicated that ANKRD22-FLAG localized peripherally on LD, and Lipi-Green stained hydrophobic core of LD.

### Both protein N-myristoylation and the membrane domain affected the localization of ANKRD22 to LD

**Figure 4:**
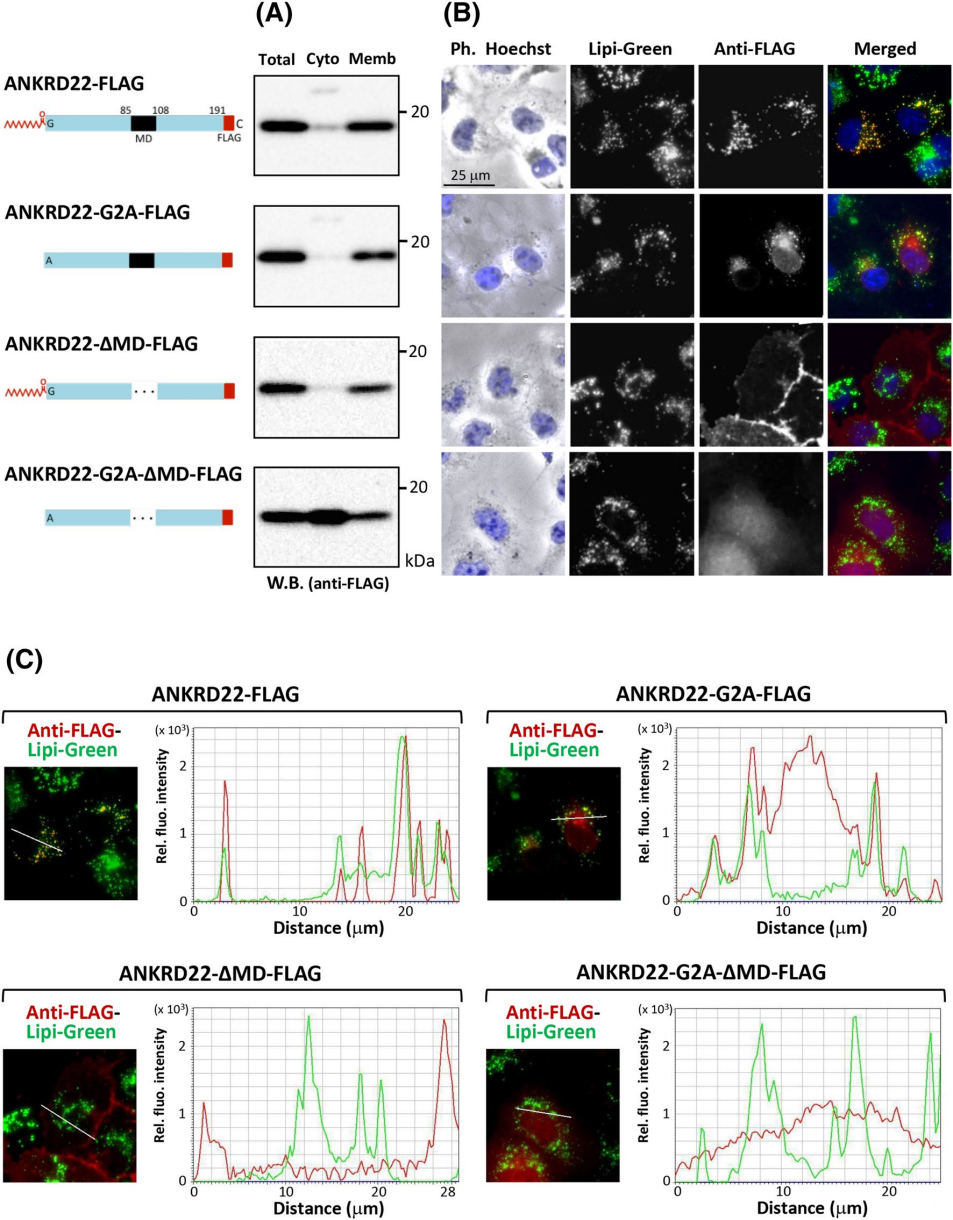
Both protein N-myristoylation and the membrane domain affected the localization of ANKRD22 to LD. Three ANKRD22-FLAG mutants, in which protein N-myristoylation and/or the putative transmembrane domain were deleted, were generated, and their membrane localization and intracellular localization were compared with those of wild-type ANKRD22-FLAG. The experiments were repeated 3 times and the similar results were obtained. A representative data was presented. (**A**) Total cell lysates of COS-1 cells transfected with cDNA encoding ANKRD22-FLAG and its three mutants were separated into cytosolic and membrane fractions using a membrane protein extraction kit (Trident). The presence of proteins in each fraction was determined by Western blotting analysis using anti-FLAG antibody. Using this membrane protein extraction kit, endogenous SAMM50, a membrane protein marker expressed in control non-transfected COS-1 cells, was detected exclusively in the total membrane fraction, whereas GAPDH, a cytosolic marker protein, was detected exclusively in the cytosolic fraction as shown in Supplementary Figure S8. A part of the blot was cropped. The uncropped raw image data for the blots are shown in Supplementary Figure S9. (**B**) Intracellular localization of ANKRD22-FLAG and its three mutants was determined by immunofluorescence analysis of COS-1 cells transfected with cDNA coding these proteins using anti-FLAG antibody. Hoechst and Lipi-Green were used as organelle markers for the nucleus and LD, respectively. (**C**) The results of the line profile analysis of the fluorescence images obtained in (**B**). For this analysis, corresponding line-scan graphs of the relative fluorescence intensities of green fluorescence (green line) and red fluorescence (red line) along the white line indicated in the merged images are shown.

In order to clarify the role of protein N-myristoylation and the putative transmembrane domain in membrane binding and localization of ANKRD22 to LD, three mutants in which protein N-myristoylation and/or the putative transmembrane domain were deleted were generated, and their membrane localization and intracellular localization were evaluated. As shown in Fig. 4A, efficient membrane localization comparable to wild-type ANKRD22-FLAG was observed with the non-myristoylatable G2A-mutant and mutant lacking the putative transmembrane domain. In contrast, significant cytosolic distribution was observed with the non-myristoylatable mutant lacking the membrane domain. These results indicated that both protein N-myristoylation and putative transmembrane domain could mediate membrane binding of ANKRD22. Regarding intracellular localization, specific localization to LD was observed only with wild-type ANKRD22-FLAG as shown in Fig. 4B. The line profile analysis of the obtained images further confirmed the specific localization of wild-type ANKRD22-FLAG to LD (Fig. 4C). In the case of ANKRD22-G2A**-**ΔMD-FLAG in which both protein N-myristoylation and the putative transmembrane domain were deleted, cytosolic distribution was observed. As for non-myristoylatable ANKRD22-G2A-FLAG, partial localization to LD was observed. Thus, protein N-myristoylation is not an absolute requirement for LD localization, but it seems likely that this modification positively affects the LD localization of ANKRD22. Regarding ANKRD22**-**ΔMD-FLAG in which the putative transmembrane domain was deleted, comparison of the phase contrast image and the immunofluorescence image of COS-1 cells expressing this mutant suggested the plasma membrane localization of this mutant as described in Supplementary Figure S4. Thus, both protein N-myristoylation and the putative transmembrane domain affected the membrane binding and specific localization of ANKRD22 to LD.

### ANKRD22 is an N-myristoylated hairpin-like monotopic membrane protein with its amino- and carboxyl termini facing the cytoplasm

**Figure 5:**
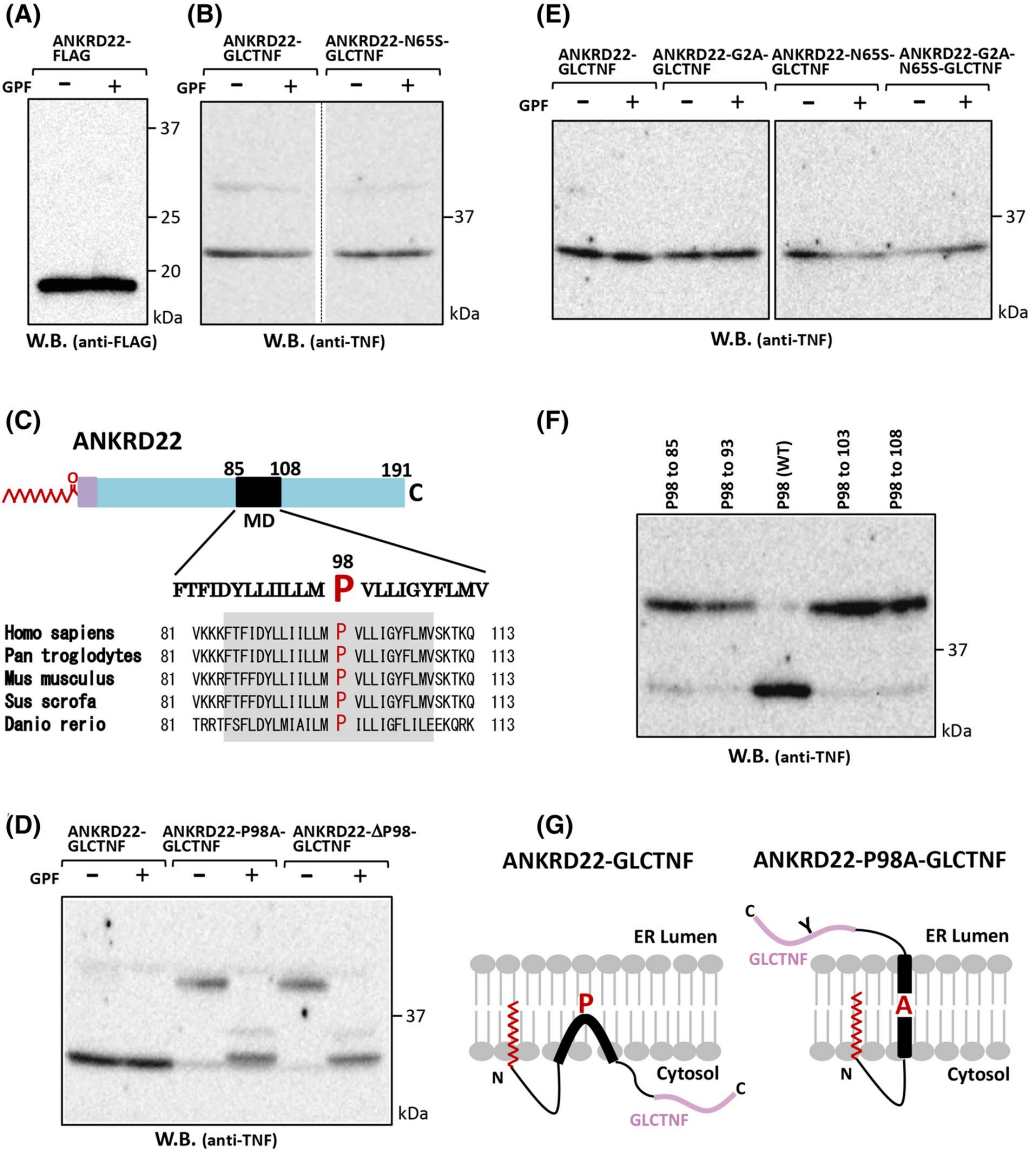
Analysis of the membrane topology of ANKRD22. The membrane topology of ANKRD22 was determined using COS-1 cells transfected with cDNA coding ANKRD22-FLAG, ANKRD22-GLCTNF, and its mutants. For this analysis, the N-glycosylation reaction occurring in the luminal domain of ER was used to assess the translocation of protein across the ER membrane. The names and structure of ANKRD22-GLCTNF and its mutants are summarized in Supplementary Figure S5. (**A**,**B**) Total cell lysates of COS-1 cells transfected with cDNA coding ANKRD22-FLAG (**A**), ANKRD22-GLCTNF, or ANKRD22-N65S-GLCTNF (**B**) were treated with or without GPF, and then subjected to Western blotting analysis using anti-FLAG or anti-TNF antibody. A part of the blot was cropped. The position of the splice is indicated by vertical dashed line in (**B**). The uncropped raw image data for the blots are shown in Supplementary Figure S10. (**C**) Amino acid sequence of the membrane domain of ANKRD22. Interspecies alignment of the membrane domain of ANKRD22 is shown. The conserved Pro residue is indicated by the red letter. (**D**,**E**) Total cell lysates of COS-1 cells transfected with cDNA coding ANKRD22-GLCTNF, ANKRD22-P98A-GLCTNF, ANKRD22-ΔP98-GLCTNF (**D**), or ANKRD22-GLCTNF, ANKRD22-G2A-GLCTNF, ANKRD22-N65S-GLCTNF, or ANKRD22-G2A-N65S-GLCTNF (**E**) were treated with or without GPF, and then subjected to Western blotting analysis using anti-TNF antibody. The raw image data for the blots are shown in Supplementary Figures S10 and S11. (**F**) Total cell lysates of COS-1 cells transfected with cDNA coding P98 to 85, P98 to 93, P98(WT), P98 to 103, or P98 to 108 were subjected to Western blotting analysis using anti-TNF antibody. The raw image data for the blot is shown in Supplementary Figure S11. (**G**) Schematic representation of the membrane topology of ANKRD22-GLCTNF and ANKRD22- P98A-GLCTNF.

As the putative transmembrane domain was demonstrated to be required for membrane binding of ANKRD22, as shown in Fig. 4, it is probable that ANKRD22 cotranslationally integrates into the ER membrane through the putative transmembrane domain. As protein N-myristoylation occurs in the cytoplasm, the N-myristoylated N-terminus is predicted to reside in the cytoplasm, suggesting that the membrane domain of ANKRD22 functions as a type II signal anchor sequence and generates a single-pass membrane protein with N-cyto/C-exo topology. To assess the membrane topology of ANKRD22, we used the N-glycosylation reaction occurring in the luminal domain of the ER^17^. The names and structure of ANKRD22 mutants analyzed in this study are summarized in Supplementary Figure S5. When the total cell lysates of COS-1 cells transfected with ANKRD22-FLAG cDNA were treated with glycopeptidase F (GPF), no change in the band pattern on Western blotting was observed, as shown in Fig. 5A, suggesting that ANKRD22 is not an N-glycosylated protein. Next, we generated ANKRD22-GLCTNF in which GLCTNF, an N-glycosylatable model protein, was fused to the C-terminus of ANKRD22. To determine whether the difference in the epitope tag attached to the C-terminus of ANKRD22 affect the LD localization, intracellular localization of tag free-ANKRD22, ANKRD22-FLAG, and ANKRD22-GLCTNF expressed in the transfected COS-1 cells were determined by immunofluorescence analysis using anti-ANKRD22 antibody. As shown in Supplementary Figure S3, all the exogenously expressed ANKRD22 constructs were specifically detected in LD irrespective of the difference in the epitope tag attached to the C-terminus. These results indicated that the difference in the epitope tag attached to the C-terminus did not affect the LD localization of ANKRD22. We next performed the analysis of membrane topology of ANKRD22-GLCTNF using the total cell lysates of COS-1 cells transfected with ANKRD22-GLCTNF.

As shown in Fig. 5B lanes 1 and 2, no change in the band pattern on Western blotting was observed after GPF treatment, suggesting that N-glycosylation did not occur on ANKRD22-GLCTNF and the C-terminal domain of ANKRD22 is located on the cytoplasmic side of the ER membrane. Thus, ANKRD22 may be a hairpin-like monotopic membrane protein with its amino- and carboxyl termini facing the cytoplasm. To confirm the retention of the N-terminal domain in the cytoplasm, an N-glycosylation site was introduced at position 63 (N63-A64-S65) of ANKRD22-GLC-TNF (ANKRD22-N65S-GLCTNF) and its susceptibility to N-glycosylation was evaluated by GPF treatment. As shown in Fig. 5B lanes 3 and 4, no change in the band pattern on Western blotting was observed after GPF treatment, suggesting that the N-terminal domain of ANKRD22 is located on the cytoplasmic side of the ER membrane.

### Pro98 plays an essential role in the formation of the hairpin-like monotopic topology of ANKRD22

The hairpin-like monotopic topology of LD protein was previously demonstrated for plant oleosins^25,26^ and the Pro residue in the hydrophobic segment was found to be required for this segment to form its hairpin-like topology^25^. Similar membrane topology was proposed for several LD proteins such as AUP1and GPAT4^27,28^. As shown in Fig. 5C, Pro98 in the hydrophobic domain of ANKRD22 is highly conserved among vertebrates, suggesting that the Pro at this position plays an important role in the formation of the hairpin-like monotopic topology. To clarify the role of Pro98 in the membrane topology formation of ANKRD22, ANKRD22-P98A-GLCTNF and ANKRD22-ΔP98-GLCTNF, in which the Pro residue in ANKRD22-GLCTNF was replaced with Ala or deleted, were generated and their membrane topology on the ER membrane was investigated based on their susceptibility to protein N-glycosylation. As shown in Fig. 5D, a significant increase in the molecular mass was observed for ANKRD22-P98A-GLCTNF and ANKRD22-ΔP98-GLCTNF, and these shifts in band patterns on SDS-PAGE were abolished by GPF treatment. Thus, the hairpin-like monotopic topology of ANKRD22 was destroyed by the lack of Pro98, the C-terminal domain of these two proteins translocated across the ER membrane, and glycosylation occurred. As such, Pro98 is essential for the formation of the hairpin-like monotopic topology of ANKRD22. Experimental results similar to those of ANKRD22-GLCTNF and ANKRD22-N65S-GLCTNF were observed when non-myristoylatable G2A-mutants of these two proteins were used, suggesting that the retention of the N-terminal domain of ANKRD22 on the cytoplasmic side does not depend on protein N-myristoylation (Fig. 5E).

To further assess the requirement of Pro98 for membrane topology formation, the effects of the position of the Pro residue in the membrane domain on the formation of hairpin-like monotopic topology of ANKRD22-GLCTNF were examined using 4 mutants in which the Pro residue was moved from position 98 to 4 different positions in the putative membrane domain. As shown in Fig. 5F, efficient N-glycosylation was similarly observed with all Pro-mutants of ANKRD22-GLCTNF; however, glycosylation was not observed with wild-type ANKRD22-GLCTNF. Therefore, the position of the Pro residue strongly affects the membrane topology of ANKRD22 and the hairpin-like monotopic topology is only formed when the Pro residue is located near the center of the membrane domain. A schematic of the membrane topology of ANKRD22-GLCTNF and ANKRD22-P98A-GLCTNF is shown in Fig. 5G.

### Both the hairpin-like monotopic topology and protein N-myristoylation affected the sorting of ANKRD22 from the ER to LD

**Figure 6:**
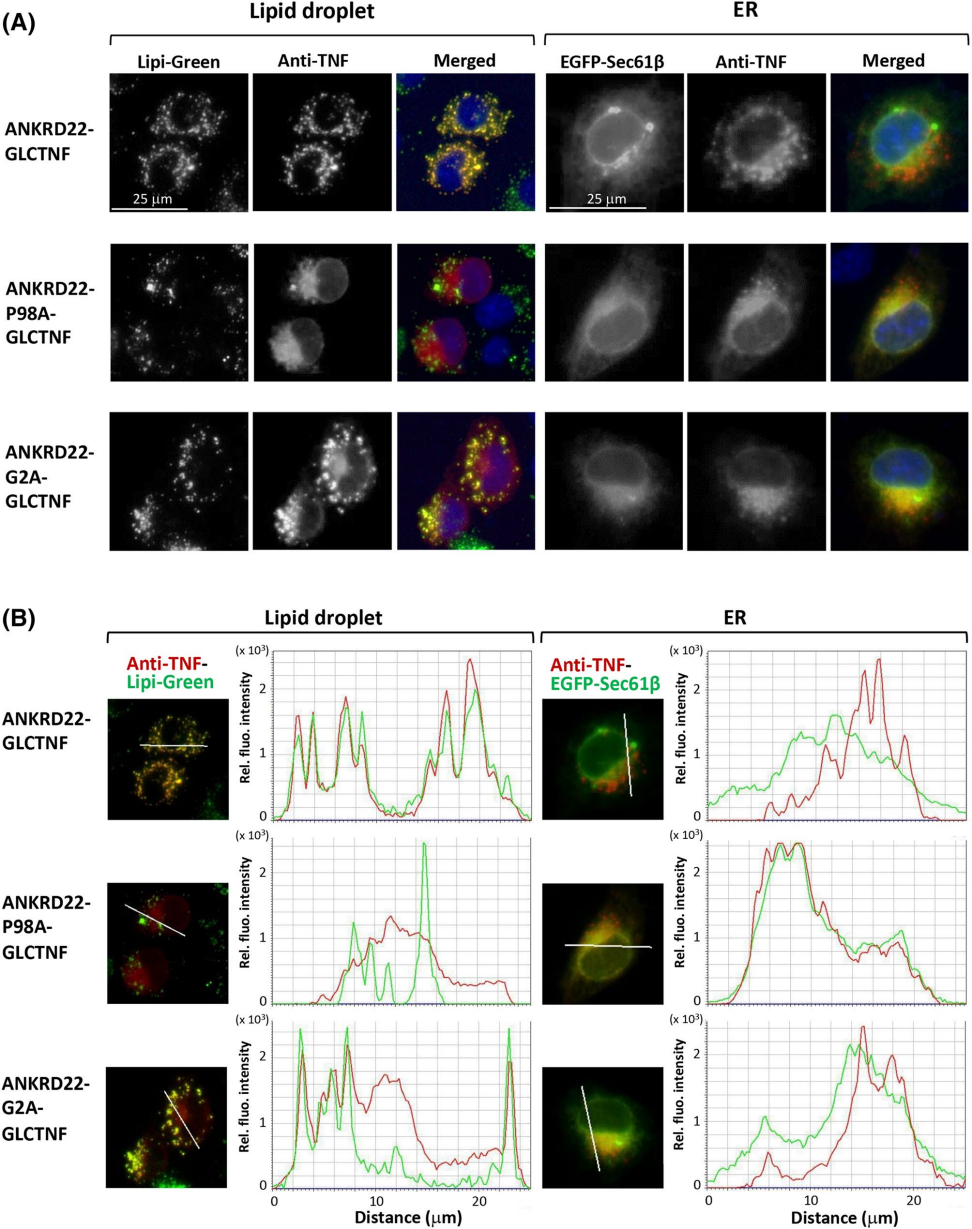
Both the hairpin-like monotopic topology and protein N-myristoylation affected the sorting of ANKRD22 from the ER to LD. The roles of the hairpin-like monotopic topology and protein N-myristoylation in the intracellular localization of ANKRD22 were examined by immunofluorescence microscopic analysis of COS-1 cells transfected with cDNA coding wild-type ANKRD22-GLCTNF or two mutants, ANKRD22-P98A-GLCTNF and ANKRD22-G2A-GLCTNF. For this analysis, Lipi-Green and EGFP-Sec61β were used as the LD marker and ER marker, respectively. The experiments were repeated 3 times and the similar results were obtained. A representative data was presented. (**A**) Fluorescence images obtained by immunofluorescence analysis. (**B**) The results of the line profile analysis of the fluorescence images obtained in (**A**). For this analysis, corresponding line-scan graphs of the relative fluorescence intensities of green fluorescence (green line) and red fluorescence (red line) along the white line indicated in the merged images are shown.

We next investigated the role of the hairpin-like monotopic topology and protein N-myristoylation in the intracellular localization of ANKRD22 by immunofluorescence microscopic analysis using wild-type ANKRD22-GLCTNF and two mutants, ANKRD22-P98A-GLCTNF and ANKRD22-G2A-GLCTNF. LD proteins with a hairpin-like topology are synthesized on the ER membrane and then sorted to LD. Therefore, for this analysis, Lipi-Green and EGFP-Sec61β were used as an LD marker and ER marker, respectively. Wild-type ANKRD22-GLCTNF specifically localized to LD, as shown in the upper left panels of Fig. 6A. In this case, localization to the ER was not observed (upper right panels). The line profile analysis of the obtained images further confirmed these observations as shown in Fig. 6B upper panels. In contrast, most ANKRD22-P98A-GLCTNF was not localized to LD, but mainly to the ER (Fig. 6A middle panels). The line profile analysis also supported these observations (Fig. 6B middle panels). As for non-myristoylatable ANKRD22-G2A-GLCTNF, partial localization to both LD and the ER was observed (Fig. 6A,B lower panels). These results suggested that the hairpin-like monotopic topology mediated by Pro98 is critical for LD localization of ANKRD22. Protein N-myristoylation is not an absolute requirement, but it positively affects the localization to LD. It was also suggested that ANKRD22 is cotranslationally integrated into the ER membrane as a hairpin-like monotopic membrane protein and then sorted to LD.

### Positively charged residues in the C-terminal region are required for localization of ANKRD22 to LD

**Figure 7:**
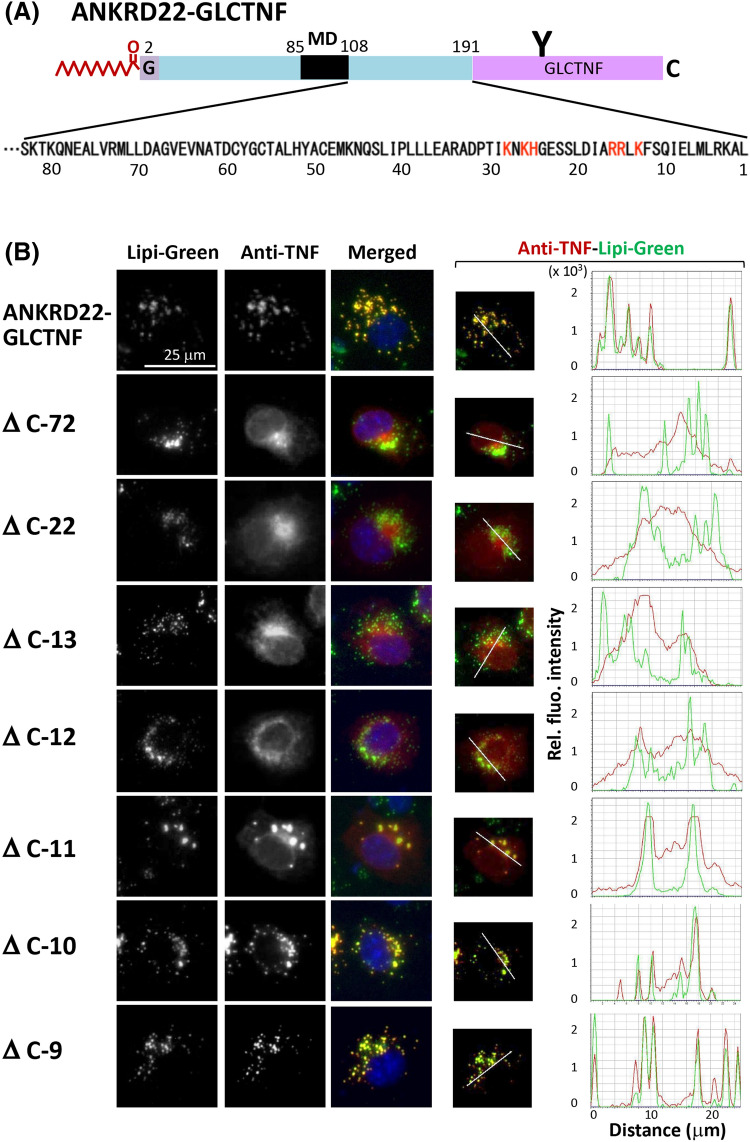
The C-terminal cytoplasmic domain plays important role in the localization of ANKRD22 to LD. The role of the C-terminal cytosolic domain in the intracellular localization of ANKRD22 was assessed by immunofluorescence microscopic analysis using a series of deletion mutants of ANKRD22-GLCTNF. The names and structure of the ANKRD22-GLCTNF mutants analyzed in Fig. 7 are summarized in Supplementary Fig. S6[1]. The experiments were repeated 3 times and the similar results were obtained. A representative data was presented. (**A**) The amino acid sequence of the C-terminal cytosolic domain of ANKRD22 was shown. Two positively charged amino acid clusters analyzed in Fig. 8. were indicated by the red characters in the amino acid sequence. (**B**) Effects of deletion of the C-terminal cytosolic domain on the intracellular localization of ANKRD22-GLCTNF. Fluorescence images obtained by immunofluorescence analysis and the results of the line profile analysis were shown. For the line profile analysis, corresponding line-scan graphs of the relative fluorescence intensities of green fluorescence (green line) and red fluorescence (red line) along the white line indicated in the merged images are shown.

**Figure 8:**
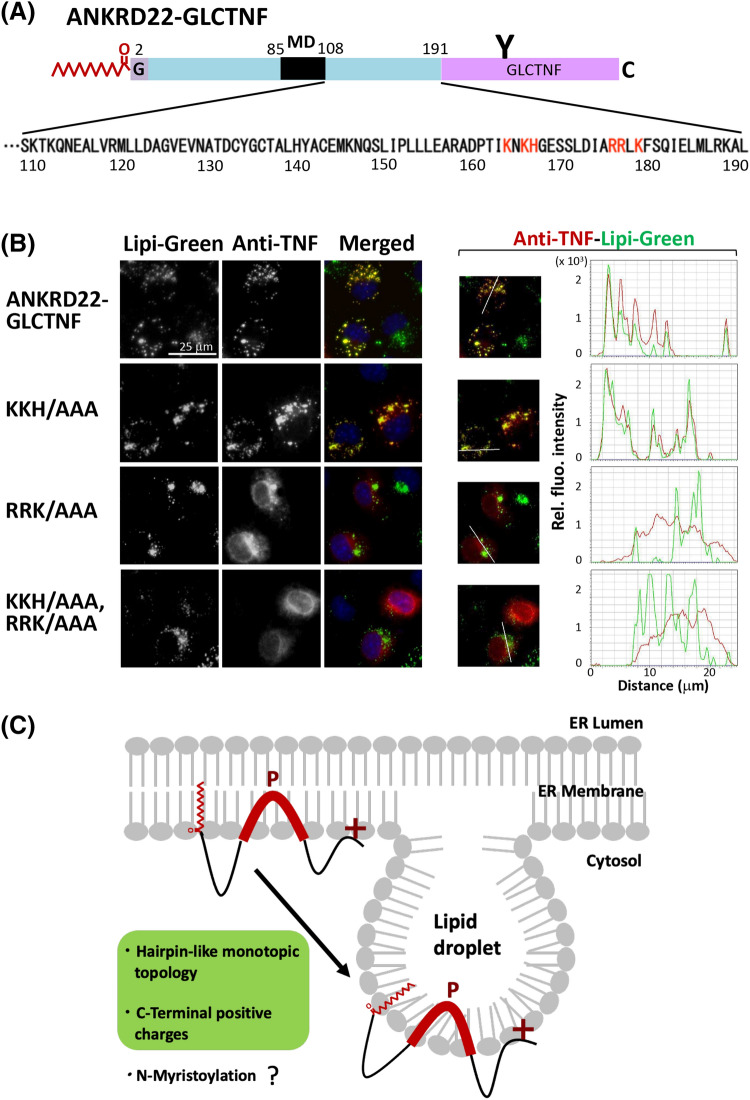
Positively charged residues in the C-terminal region are required for localization of ANKRD22 to LD. The role of the C-terminal cytosolic domain in the intracellular localization of ANKRD22 was assessed by immunofluorescence microscopic analysis using a series of substitution mutants of ANKRD22-GLCTNF. The names and structure of the ANKRD22-GLCTNF mutants analyzed in Fig. 8 are summarized in Supplementary Fig. S6[2]. The experiments were repeated 3 times and the similar results were obtained. A representative data was presented. (**A**) The amino acid sequence of the C-terminal cytosolic domain of ANKRD22 was shown. Two positively charged amino acid clusters were indicated by the red characters in the amino acid sequence. (**B**) Effects of substitution of the C-terminal positive-charge clusters on the intracellular localization of ANKRD22-GLCTNF. Fluorescence images obtained by immunofluorescence analysis and the results of the line profile analysis were shown. For the line profile analysis, corresponding line-scan graphs of the relative fluorescence intensities of green fluorescence (green line) and red fluorescence (red line) along the white line indicated in the merged images are shown. (**C**) The role of the hairpin-like monotopic membrane topology, C-terminal positively charged residues, and protein N-myristoylation on the LD localization of ANKRD22. Multiple factors, including the hairpin-like monotopic membrane topology, C-terminal positively charged residues, and protein N-myristoylation cooperatively affect the intracellular targeting of ANKRD22 from the ER to LD. Among them, the hairpin-like monotopic membrane topology and C-terminal positively charged residues are required for proper targeting of ANKRD22 to LD. As for protein N-myristoylation, it positively affects the LD localization. However, the exact roles of protein N-myristoylation in the intracellular localization and function of ANKRD22 are not clear.

We demonstrated that the hairpin-like monotopic membrane domain and protein N-myristoylation affected the localization of ANKRD22 to LD, as shown in Figs. 4, 5 and 6. To clarify other structural determinants of ANKRD22 for LD localization, we next examined the role of the C-terminal cytosolic domain in the intracellular localization of ANKRD22. For this purpose, a series of deletion mutants of ANKRD22-GLCTNF, in which the 9–72 C-terminal residues of ANKRD22 in ANKRD22-GLCTNF were deleted, were constructed, and their intracellular localization was examined by immunofluorescence microscopic analysis. The amino acid sequence of the C-terminal cytosolic domain of ANKRD22 was shown in Fig. 7A. The structure of each deletion mutant is shown in Supplementary Fig. S6[1]. As a result, truncation of the C-terminal domain strongly affected the intracellular localization of ANKRD22. The deletion of 9 C-terminal residues (Δ C-9) did not affect the localization, as shown in the bottom panels in Fig. 7B. However, localization to LD was significantly reduced as the number of deleted amino acid residues increased from 10 to 12, and localization to LD disappeared when 13 C-terminal residues were deleted (Δ C-13). The line profile analysis of the obtained images further confirmed these observations as shown in Fig. 7B right panels. Two positively charged amino acid clusters, KNKH at position 164 to 167 and RRLK at 176 to 179, are present close to the deleted C-terminal region as indicated by the red characters in the amino acid sequence of the C-terminal region (Fig. 8A). As it was previously reported that positively charged residues in the cytoplasmic domain of hairpin-like monotopic protein, such as AUP1, play important role in the localization to LD^27^, we investigated the role of these two positive charge clusters in the intracellular localization of ANKRD22. For this purpose, three ANKRD22-GLCTNF mutants, in which positively charged residues in either or both of the two C-terminal positive charge clusters were changed to Ala, were constructed (Supplemental Fig. S6[2]) and their intracellular localization was determined by immunofluorescence microscopic analysis. As a result, a positive charge cluster RRLK at position 176 to 179 strongly affected the localization of ANKRD22, whereas another cluster KNKH at 164 to 167 did not significantly affect the localization as shown in Fig. 8B left panels. These observations were confirmed by the line profile analysis of the obtained images as shown in Fig. 8B right panels. Thus, the positively charged amino acid residues located at position 176 to 179 in the C-terminal cytoplasmic domain plays critical role in the localization of ANKRD22 to LD. As such, multiple factors, including the hairpin-like monotopic membrane topology, C-terminal positively charged residues, and protein N-myristoylation, cooperatively affected the intracellular targeting of ANKRD22 to LD (Fig. 8C).

## Discussion

Protein N-myristoylation occurs mainly on eukaryotic soluble cytoplasmic proteins, but few integral membrane proteins have been shown to be N-myristoylated thus far. Mammalian NADH-cytochrome b5 reductase 3 (CYB5R3) is an example of a eukaryotic N-myristoylated integral membrane protein. This protein is a single-pass membrane protein that is dually targeted to the outer membranes of mitochondria and the ER^29,30^. In CYB5R3, it was reported that protein N-myristoylation plays important role in sorting of this protein to the mitochondria since a non-myristoylatable G2A-mutant exclusively localizes to the ER^31,32^. In our attempt to identify novel human N-myristoylated proteins from human cDNA clones in human cDNA resources^12^, we recently identified two mitochondrial outer membrane proteins, SAMM50 and TOMM40, as N-myristoylated integral membrane proteins^16^. Regarding SAMM50, protein N-myristoylation is required for mitochondrial targeting. However, protein N-myristoylation is dispensable for the localization of TOMM40 to mitochondria. Thus, protein N-myristoylation does not necessarily function as a mitochondrial targeting signal for N-myristoylated membrane proteins. Indeed, using the same strategy, we identified another mammalian integral membrane protein, Lunapark, as an N-myristoylated double-pass integral membrane protein specifically localized to the ER^17^. Lunapark localizes mainly to the peripheral ER and induces the formation of large polygonal tubular structures when overexpressed in human cells. The morphological change in the ER induced by Lunapark was significantly inhibited by the inhibition of protein N-myristoylation, suggesting that protein N-myristoylation plays an essential role in the ER morphological change induced by Lunapark^17^. The role of Lunapark in the formation of ER morphology has been examined, and ER three-way junctions were reported to form through atlastin (ATL)-mediated membrane fusion and stabilization by Lunapark^33,34^. As for the role of protein N-myristoylation in the function of Lunapark, it was recently reported that protein N-myristoylation is required for its localization to these three-way junctions and for the selective interaction with ATLs at three-way junctions^35^. Based on these studies, protein N-myristoylation can mediate diverse roles in the intracellular localization and function of N-myristoylated integral membrane proteins.

In this study, we searched for novel human N-myristoylated transmembrane proteins from human proteins listed in the UniProtKB/Swiss-Prot protein database using a strategy established in our previous studies and found that ANKRD22 is an N-myristoylated hairpin-like monotopic membrane protein with its amino- and carboxyl termini facing the cytoplasm that specifically localizes to LD. LD are fat storage organelles present in most eukaryotic cells^36^. In LD, the neutral lipid core containing triglycerides, sterol esters and other lipophilic molecules is covered by a monolayer of phospholipids, in which numerous proteins are embedded. Many of the proteins specifically localizing to the phospholipid monolayer surface of LD mediate important metabolic and signaling functions^37^.

How proteins specifically localize to the LD is under investigation. A wide variety of proteins has been identified on LD; however, no specific LD localization signals have been found. Many LD proteins can access the LD surface by one of two major pathways, one from the ER and one from the cytosol. The former pathway was designated as class I and the latter as class II^38^. Class I LD proteins often have a membrane-embedded, hydrophobic ‘hairpin’ motif that is flanked by cytosolic hydrophilic N- and C-termini, and access LD from the ER. The class I proteins are cotranslationally embedded into the cytoplasmic leaflet of the ER membrane during synthesis on the translocation channel (translocon) of the ER and then targeted to LD^39^. Examples of class I hairpin proteins that traffic from the ER to LD include AUP1, caveolin-1, DGAT2, GPAT4, and UBXD8^39^. As shown in Fig. 5, ANKRD22 is a hairpin-like monotopic membrane protein with its amino- and carboxyl termini facing the cytoplasm, suggesting it to be a class I LD protein. A common feature of these hydrophobic hairpin domains is a conserved Pro residue positioned in the center of the hydrophobic region. This Pro residue was hypothesized to break the helix and generate a kink, resulting in a hairpin conformation. With this hairpin topology, both ends of the hydrophobic domain face the cytosol on the ER or on LD. In ANKRD22, Pro98 in the center of the hydrophobic domain of ANKRD22 is highly conserved among vertebrates (Fig. 5C), suggesting that the Pro at this position plays an important role in the formation of the hairpin-like monotopic topology. Indeed, mutation or deletion of Pro98 strongly affected the membrane topology of ANKRD22 and converted it to a single-pass transmembrane protein (Fig. 5D). The position of the Pro residue in the hydrophobic domain strongly affected the topology of ANKRD22 and the hairpin conformation was generated only when the Pro residue was positioned at the center of the membrane domain (Fig. 5F). Moreover, trafficking of ANKRD22 from the ER to LD was prevented by this conversion of membrane topology (Fig. 6). Thus, Pro98 is essential for determining the membrane topology and trafficking of ANKRD22. Similar experiments demonstrating the important roles of the Pro residue in the hydrophobic domain of LD proteins in determining the hairpin-like topology and trafficking to LD have been reported for several LD proteins such as AUP1 and caveolin-1^27,40^.

Although the hydrophobic hairpin region of class I LD proteins is generally necessary and sufficient for LD targeting, trafficking of some proteins from the ER to LD is affected by other structural factors such as flanking positively-charged residues or an amphipathic helix located N-terminally of the hydrophobic region^41,42^. As for ANKRD22, protein N-myristoylation positively affected the localization of this protein to LD (Fig. 4B,C). Protein N-myristoylation was demonstrated to function as a membrane anchor. Indeed, N-myristoylated ANKRD22 mutants, in which the hydrophobic membrane domain was deleted, localized to the plasma membrane, whereas the non-myristoylatable forms exhibited cytosolic localization, suggesting that protein N-myristoylation alone can function as an efficient membrane anchor of ANKRD22 (Fig. 4). Thus, membrane anchoring of the N-terminus of ANKRD22 mediated by protein N-myristoylation can positively affect the targeting to LD. In this case, however, protein N-myristoylation is not absolutely required for LD localization because a part of non-myristoylatable G2A mutant localized to LD. Regarding the role of protein lipid modification in the targeting of LD proteins to LD, protein palmitoylation and protein prenylation were reported to affect class II LD proteins. For example, the Arf-GAP ELMOD2 is palmitoylated^43^ and aldehyde dehydrogenase ALDH3B2 is prenylated with a geranylgeranyl group^44^. Both proteins require lipid modification for LD targeting. Another example of a protein that utilizes a lipid anchor to target LD is the small GTPase Rab18. Rab18 contains a mono-cysteine prenylation motif (CAAX motif) in its C-terminus^45^. Thus, lipid modification positively affected the LD targeting of class II LD proteins. However, the role of lipid modification in the targeting of class I LD proteins to LD is not well understood. The present study suggested that protein N-myristoylation positively affect the localization of ANKRD22 to LD. In this case, however, protein N-myristoylation is not sufficient to target ANKRD22 specifically to LD, and the hairpin-like monotopic topology and C-terminal positively charged residues are also required. The role of C-terminal positively charged residues in LD targeting is not clear, but they may mediate interactions with specific negatively charged lipid headgroups or proteins in the LD monolayer. Thus, multiple factors, including the hairpin-like monotopic membrane topology, C-terminal positively charged residues, and protein N-myristoylation, cooperatively affect the intracellular targeting of ANKRD22 to LD.

ANKRD22 is a functionally unknown protein, but it was recently reported to be overexpressed in malignant cancers such as non-small cell lung cancer, colorectal cancer, and breast cancer^21–23^. In these cancer cells, ANKRD22 may exert pro-tumor effects. However, ANKRD22 suppressed the development of prostate cancer in one study^46^. Thus, the effects of ANKRD22 on cancer may differ among cancer types. In non-small cell lung cancer, ANKRD22 up-regulated the transcription of E2F1 and promoted the progression of cancer cells by enhancing cell proliferation^21^. In colorectal cancer-initiating cells (CCICs), the expression of ANKRD22 was induced by the p38/MAX pathway activated by tumor microenvironment stimuli^22^. In CCICs, ANKRD22 cooperated with the lipid transport protein, Extended Synaptotagmin-1 (E-Syt1), to transport excess lipids into mitochondria and reduced the number of mitochondria in an autophagy-independent manner. In this report, ANKRD22 was identified as a mitochondrial membrane protein, but not a LD membrane protein^22^. The reason for the discrepancy between this study and ours is not clear. In breast cancer tissues, ANKRD22 promoted breast cancer cell malignancy by activating the Wnt/β-catenin pathway by regulating nucleolar and spindle-associated protein 1 (NuSAP1) expression^23^. Thus, in many types of malignant cancers, ANKRD22 may play important roles in the procession of progression of cancer through several mechanisms. However, the role of LD localization in cancer progression mediated by ANKRD22 is unknown. As LD accumulation and catabolism are tightly coupled to energetic metabolism and cell signaling, and are required for cancer cell proliferation, resistance to death, and aggressiveness^47^, it is possible that ANKRD22 specifically localizing to LD affects the status of cancer. Further studies are required to clarify the physiological function of ANKRD22 and the role of LD localization of this protein in the progression or suppression of cancer.

## Methods

### Materials

Human cDNA (Flexi ORF clones) was purchased from Promega (Madison, WI, USA). Synthetic cDNA coding ANKRD22 was obtained from GENEWIZ (Suzhou, China). Transdirect insect cell, an insect cell-free protein synthesis system, was obtained from Shimadzu (Kyoto, Japan). The Trident Membrane Protein Extraction Kit was from GeneTex (Irvine, CA, USA)^16^. The T7-Scribe standard RNA IVT kit was from CELLSCRIPT (Madison, WI, USA). [^3^H]leucine, [^3^H]myristic acid, and ECL prime Western blotting detection reagent were from GE Healthcare (Buckinghamshire, UK). ENLIGHTENING was from PerkinElmer (Waltham, MA, USA). The dye terminator cycle sequencing kit, Lipofectamine LTX and Plus reagents, MitoTracker Red CMXRos, and Hoechst 33,342 were from Life Technologies Corporation (Carlsbad, CA, USA). Lipi-Green was from Dojindo Laboratories (Kumamoto, Japan). Anti-FLAG monoclonal antibody, anti-ANKRD22 antibody, anti-SAMM50 polyclonal antibody, anti-GAPDH polyclonal antibody, anti-rabbit IgG-FITC antibody, and Nile Red were from Sigma (St. Louis, MO, USA). Anti-human TNF polyclonal antibody was from R&D Systems (Minneapolis, USA). 13-tetradecynoic acid (Alk-Myr) was from Cayman (Ann Arbor, MI, USA). Azide TAMRA (Az-TAMRA) was from Click Chemistry Tools (Scottsdale, AZ, USA). Tris(2-carboxyethyl)phosphine hydrochloride (TCEP) and tris[(1-benzyl-1*H*-1,2,3-triazol-4-yl)methyl]amine (TBTA) were from Sigma (St. Louis, MO, USA). Protein G-HRP conjugate was from Bio-Rad (Hercules, CA, USA). Glycopeptidase F was from Takara Shuzo (Kyoto, Japan). X-ray film was from Eastman Kodak (Rochester, NY, USA). The other reagents used were from Wako Pure Chemical (Osaka, Japan) or Daiichi Pure Chemicals (Tokyo, Japan) and were of analytical or DNA grade.

### Prediction of protein N-myristoylation using prediction programs

Two public WWW-server-based prediction programs for protein N-myristoylation, MYR Predictor (http://mendel.imp.ac.at/myristate/SUPLpredictor.htm)^18^ and Myristoylator (http://www.expasy.org/tools/myristoylator/)^19^, were used to predict protein N-myristoylation. The entire amino acid sequences deduced from the nucleotide sequences of the ORFs were used as the query.

### Prediction of the transmembrane domain of membrane proteins using prediction programs

A public WWW-server-based prediction program for membrane proteins, SOSUI, was used to predict the transmembrane domain of membrane proteins^20^.

### Plasmid construction

Nucleotide sequences of oligonucleotides used for plasmid construction are summarized in Supplemental Tables S1, S2, and S3. The plasmids pTD1 tGelsolin-FLAG and pTD1-Δ10aa-tGellsolin-FLAG were constructed as previously described^14^. pTD1 plasmids containing the cDNA coding tGelsolin fusion proteins with the 10 N-terminal amino acids of the proteins listed in the UniProtKB/Swiss-Prot protein database at the N-terminus were constructed using the oligonucleotides listed in Table S1 and plasmid pTD1-Δ10aa-tGellsolin-FLAG, as previously described^14^. The pcDNA3 plasmids including cDNA coding tag free-ANKRD22, FLAG- or GLCTNF-tagged ANKRD22 and its mutants were constructed by PCR or site-directed mutagenesis, as summarized in Supplementary Tables S4 and S5. The plasmids pcDNA3-FLAG and pcDNA3-GLCTNF were constructed as previously described^17^. The plasmids pcDNA3-EGFP-Sec61β and pcDNA3-TGN46-EGFP were constructed as previously described^17,48^.

### Detection of protein N-myristoylation using an insect cell-free protein synthesis system

cDNA was subcloned into the vector pTD1 (Shimadzu Co.) at a site under the control of the T7 promoter. The mRNA encoding the cDNA was prepared using an AmpliScribe T7 High Yield Transcription Kit according to the manufacturer’s instructions. The translation reaction was carried out using an insect cell-free protein synthesis system (Shimadzu Co.) in the presence of [^3^H]leucine or [^3^H]myristic acid under conditions recommended by the manufacturer. The mixture (composed of 12.5 μL of insect cell lysate, 7.5 μL of reaction buffer, 0.5 μL of 1 mM leucine-free amino acid mixture, 2.0 μL of [^3^H]leucine (2 μCi) or [^3^H]myristic acid (40 μCi), and 2.5 μL of mRNA (5 μg)) was incubated at 25 °C for 6 h^49^. The samples were then analyzed by SDS–PAGE and fluorography.

### Transfection of cells

COS-1 (simian virus 40-transformed African green monkey kidney cell line, American Type Culture Collection) cells and HepG2 (human hepatocellular carcinoma cell line, American Type Culture Collection) cells were maintained in Dulbecco’s modified Eagle’s medium (DMEM; Gibco BRL [Palo Alto, CA, USA]) supplemented with 10% fetal calf serum (FCS; Gibco BRL). Cells (2 × 10^5^) were plated onto 35-mm diameter dishes 1 day before transfection. pcDNA3 constructs (2 μg) containing cDNA encoding FLAG-tagged or GLCTNF-fusion proteins were transfected into the cells in each plate using 2.5 μL of Lipofectamine LTX and 2 μL of Plus reagent in 1 mL of serum-free medium^16^. After incubation for 5 h at 37 °C, serum-containing medium was added and cells were incubated again at 37 °C for appropriate periods.

### Metabolic labeling of cells

Metabolic labeling of cells with the myristic acid analog (Alk-Myr) was performed as described previously^16^. Cells (2 × 10^5^) were transfected with pcDNA3 constructs (2 μg) containing cDNA as described above and incubated at 37 °C for 12 h. Then, they were washed once with 1 mL of serum-free DMEM and incubated for 10 h at 37 °C in 1 mL of DMEM (+ 2% FCS) containing 25 μM Alk-Myr. Subsequently, the cells were washed three times with Dulbecco’s phosphate-buffered saline (DPBS), harvested, and lysed with 200 μL of RIPA buffer (50 mM Tris–HCl (pH 7.5), 150 mM NaCl, 1% Nonidet P-40, 0.5% sodium deoxycholate, 0.1% SDS, and protease inhibitors) on ice for 20 min. The samples labeled with Alk-Myr were reacted with Az-TAMRA by click chemistry and protein N-myristoylation was analyzed by in-gel fluorescence analysis.

### Cu(I)-catalyzed azide-alkyne cycloaddition (CuAAC)

The cell lysates labeled with Alk-Myr (46 µL) were reacted with 4 µL of freshly premixed click chemistry reaction cocktail (1 µL Az-TAMRA [5 mM], 1 µL TCEP [50 mM], 1 µL TBTA [5 mM], and 1 µL CuSO_4_·5H_2_O [50 mM]) in a total reaction volume of 50 µL for 1 h at room temperature. After CuAAC, 500 µL of MeOH was added to the samples and they were placed at − 80 °C overnight. After centrifugation at 15,000 rpm at 4 °C for 30 min, the supernatant was removed. Thereafter, the pellet was washed with 500 µL of MeOH and air dried^16^. The samples were denatured by sonication in SDS-sample buffer and then subjected to SDS-PAGE. In-gel fluorescence analysis of SDS-PAGE gel was performed using Typhoon FLA9500 (GE-Healthcare Bio-Sciences AB, Uppsala, Sweden)^50^.

### Determination of N-glycosylation of proteins

To examine the N-glycosylation of GLC-TNF-fusion proteins, 24 h after transfection, total cell lysates were obtained as described above and 20 μL of the total cell lysate of each group of transfected cells was treated with 10 mU/mL of GPF at 37 °C for 1 h, and then analyzed by Western blotting^17^.

### SDS-PAGE and fluorography

The radiolabeled proteins were resolved by 12.5% SDS-PAGE, and the gel was fixed and soaked in ENLIGHTENING (PerkinElmer) for 20 min. Thereafter, the gel was dried under a vacuum and exposed to X-ray film for an appropriate period.

### Western blotting

Proteins were resolved by 12.5% SDS-PAGE and then transferred to an Immobilon-P transfer membrane. After blocking with non-fat milk, the membrane was probed with a primary antibody, as described previously^51^. Immunoreactive proteins were detected specifically by incubation with protein G-HRP conjugate. The membrane was developed using ECL Prime Western blotting detection reagent and bands were detected using a MicroChemi Chemiluminescence Imaging System (Berthold Technologies, Bad Wildbad, Germany).

### Isolation of total membrane proteins and cytosolic proteins in cells

Isolation of total membrane proteins and cytosolic proteins in intact or transfected COS-1 cells was performed using the Trident Membrane Protein Extraction Kit (Gene Tex) according to the manufacturer’s protocol^16^. The obtained total membrane protein fraction and cytosolic fraction were subjected to Western blotting analysis.

### Immunofluorescence analysis and fluorescence microscopy

Immunofluorescence analysis of transfected cells was performed 24 h after transfection^52^. After staining with Hoechst 33342 and MitoTracker Red, Nile Red, or Lipi-Green, the cells were washed with DPBS, fixed in 4% paraformaldehyde in DPBS for 15 min, and permeabilized with 0.1% Triton X-100 in DPBS for 10 min at room temperature, followed by washing with 0.1% gelatin in DPBS. The permeabilized cells were incubated with a specific antibody in DPBS for 1 h at room temperature. After washing with 0.1% gelatin in DPBS, the cells were incubated with anti-IgG-FITC or anti-IgG-ALEXA594 antibody for 1 h at room temperature. The concentration of fluorescent probes and 1st- and 2nd-antibodies used in the immunofluorescence analysis was summarized in supplementary Table S6. After washing with 0.1% gelatin in DPBS, the cells were observed using a Leica AF7000 fluorescence microscope (Leica, Solmser, Germany). Line profile analysis was performed using the Line Profile Tool of Leica LAS AF software (Leica, Solmser, Germany). For this analysis, the white lines in merge panels were converted to line profiles using the Line Profile Tool.

## Supplementary Information

Supplementary Information.
