# Supplementary material for: ANKRD22 is an N-myristoylated hairpin-like monotopic membrane protein specifically localized to lipid droplets
Source: Sci Rep. 2021 Sep 28;11:19233. doi: 10.1038/s41598-021-98486-8 (PMC8478909; doi:10.1038/s41598-021-98486-8)
Supplement: Supplementary file 1 — Supplementary Information. [file 41598_2021_98486_MOESM1_ESM.pdf]

Supplementary Information for

ANKRD22 is an N-myristoylated hairpin-like monotopic membrane protein specifically localized to lipid droplets

Toshihiko Utsumi[1, 2]*, Takuro Hosokawa[1], Mayu Shichita[1], Misato Nishiue[1], Natsuko Iwamoto[1], Haruna Harada[1], Aya Kiwado[1], Manami Yano[1], Motoaki Otsuka[1], & Koko Moriya[1]

[1] Graduate School of Sciences and Technology for Innovation, Yamaguchi University, Yamaguchi 753-8515, Japan

[2] Department of Biological Chemistry, Faculty of Agriculture, Yamaguchi University, Yamaguchi 753-8515, Japan

*Corresponding author

e-mail: utsumi@yamaguchi-u.ac.jp

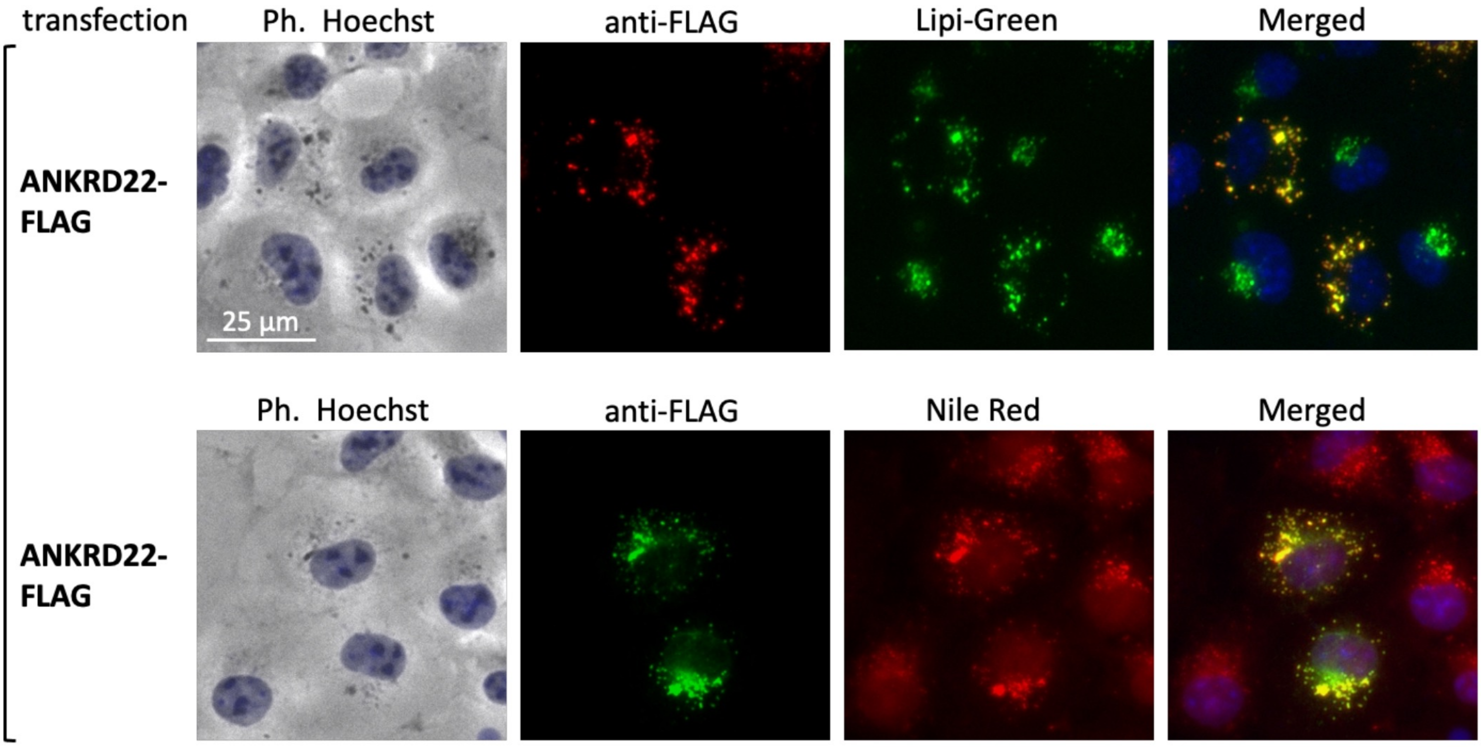

Supplementary Figure S1

Identification of intracellular localization of ANKRD22-FLAG expressed in COS-1 cells by Nile Red staining.

To confirm the specific localization of ANKRD22 to LD, COS-1 cells transfected with ANKRD22-FLAG cDNA were stained with Lipi-Green (0.1 μM) or Nile ReD (0.2 μg/ml). Thereafter, the intracellular localization of ANKRD22-FLAG was assessed by immunofluorescence analysis using anti-FLAG antibody. The experiments were repeated 3 times and the similar results were obtained. A representative data was presented. As shown in the upper panels, the fluorescence signals of ANKRD22-FLAG were efficiently merged with Lipi-Green fluorescence. As for Nile red staining, similar efficient colocalization of fluorescence signals were observed despite the presence of high background of Nile Red staining as shown in the lower panels. From these results, it is suggested that ANKRD22-FLAG expressed in COS-1 cells specifically localized to LD.

Fig. S1.

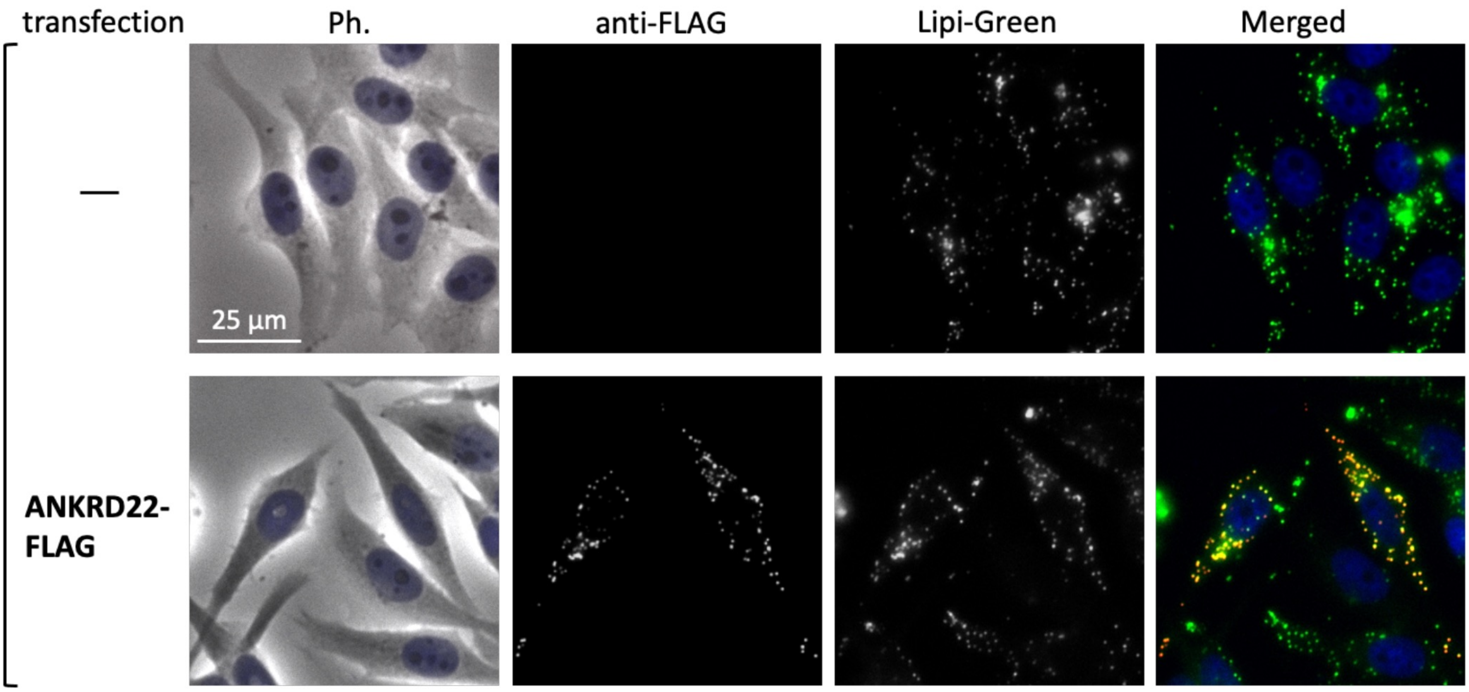

Supplementary Figure S2

ANKRD22-FLAG expressed in human HepG2 cells specifically localized to LD.

To determine the localization of ANKRD22 in human cells, human HepG2 cells transfected with ANKRD22-FLAG cDNA were stained with Lipi-Green, then the intracellular localization of ANKRD22-FLAG was assessed by immunofluorescence analysis using anti-FLAG antibody. The experiments were repeated 3 times and the similar results were obtained. A representative data was presented. As shown in the upper panels, LD in non-transfected HepG2 cells were visualized by Lipi-Green staining. In HepG2 cells transfected with ANKRD22-FLAG, the fluorescence signals of ANKRD22-FLAG were efficiently merged with Lipi-Green fluorescence as shown in the lower panels. These results indicated that ANKRD22-FLAG expressed in human cells specifically localized to LD.

Fig. S2.

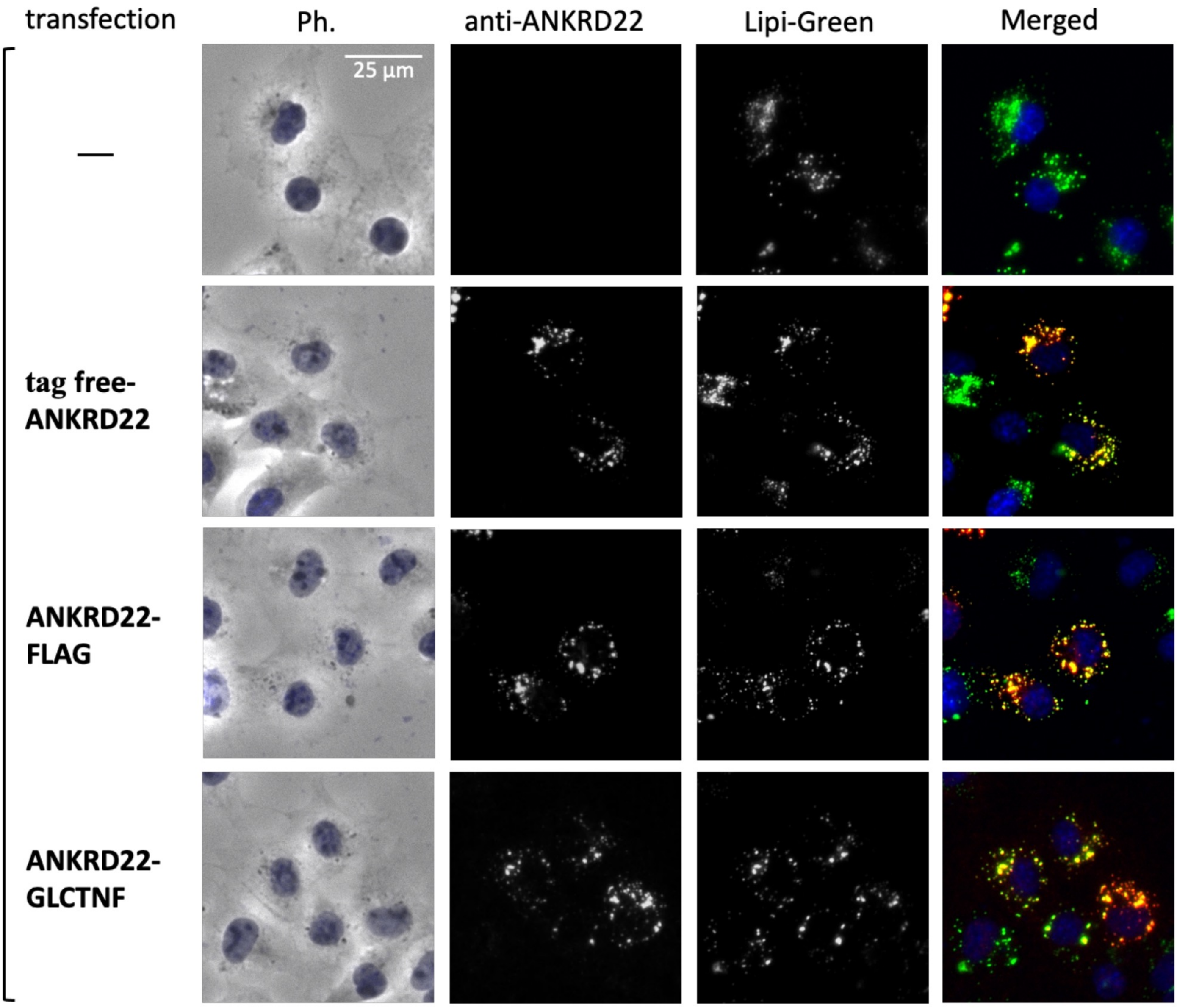

Supplementary Figure S3

Analysis of intracellular localization of endogenous ANKRD22, tag free-ANKRD22, ANKRD22-FLAG, and ANKRD22-GLCTNF expressed in COS-1 cells by immunofluorescence analysis.

To determine the intracellular localization of endogenous ANKRD22 expressed in COS-1 cells, non-transfected COS-1 cells were stained with Lipi-Green, then the intracellular localization of ANKRD22 was assessed by immunofluorescence analysis using anti-ANKRD22 antibody. As shown in the top panels, the fluorescence signal of ANKRD22 was not observed, indicating that ANKRD22 was not efficiently expressed in COS-1 cells. We next determined the intracellular localization of tag free-ANKRD22, ANKRD22-FLAG, ANKRD22-GLCTNF expressed in the transfected COS-1 cells using the same method. As shown in the lower panels, all the exogenously expressed ANKRD22 constructs were specifically detected in LD irrespective of the difference in the epitope tag attached to the C-terminus. These results indicated that the difference in the epitope tag attached to the C-terminus did not affect the LD localization of ANKRD22.

Fig. S3.

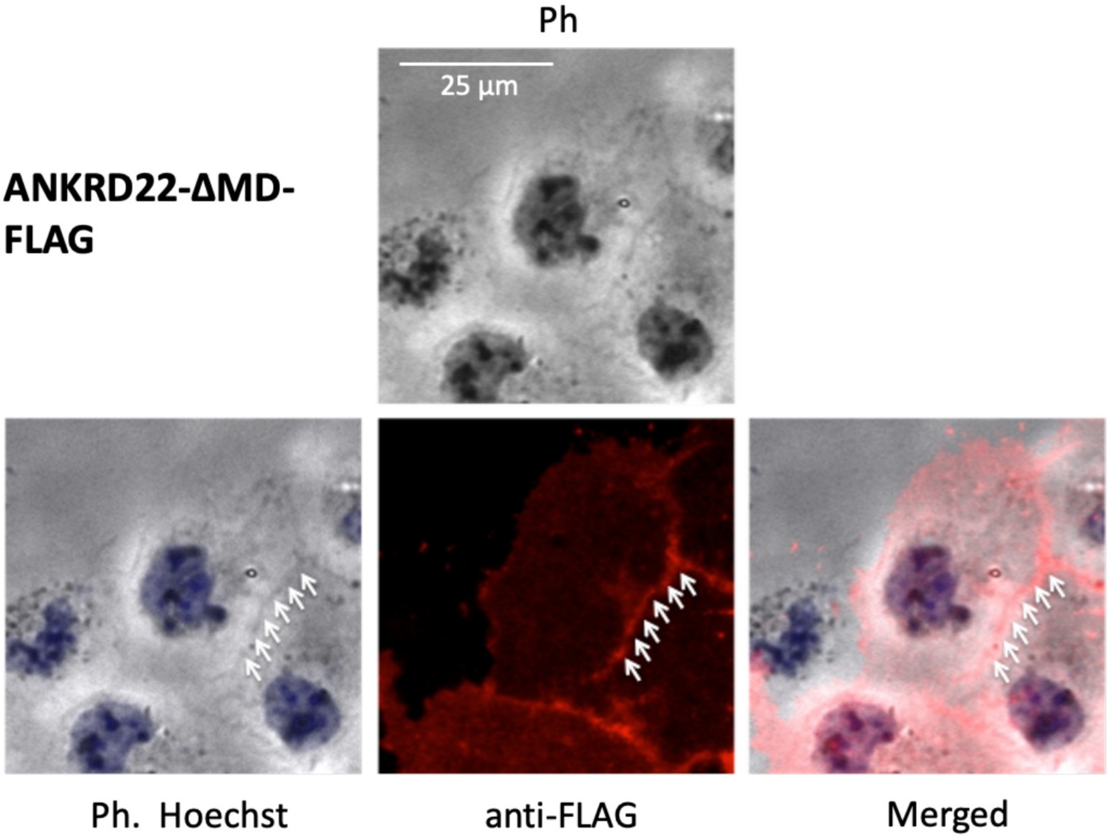

Supplementary Figure S4

Analysis of intracellular localization of ANKRD22-ΔMD-FLAG expressed in COS-1 cells by immunofluorescence analysis.

To determine the intracellular localization of ANKRD22-ΔMD-FLAG expressed in COS-1 cells, phase contrast image and immunofluorescence image of COS-1 cells expressing ANKRD22-ΔMD-FLAG used in Fig. 4B were compared. As shown in the lower left panel, the plasma membrane was detected as a boundary between the two adjacent cells as indicated by white arrows. When immunofluorescence image of ANKRD22-ΔMD-FLAG was merged with the phase contrast image, the fluorescence signal was detected on the boundary between the two cells (lower right panel), suggesting that ANKRD22-ΔMD-FLAG localized to the plasma membrane.

Fig. S4.

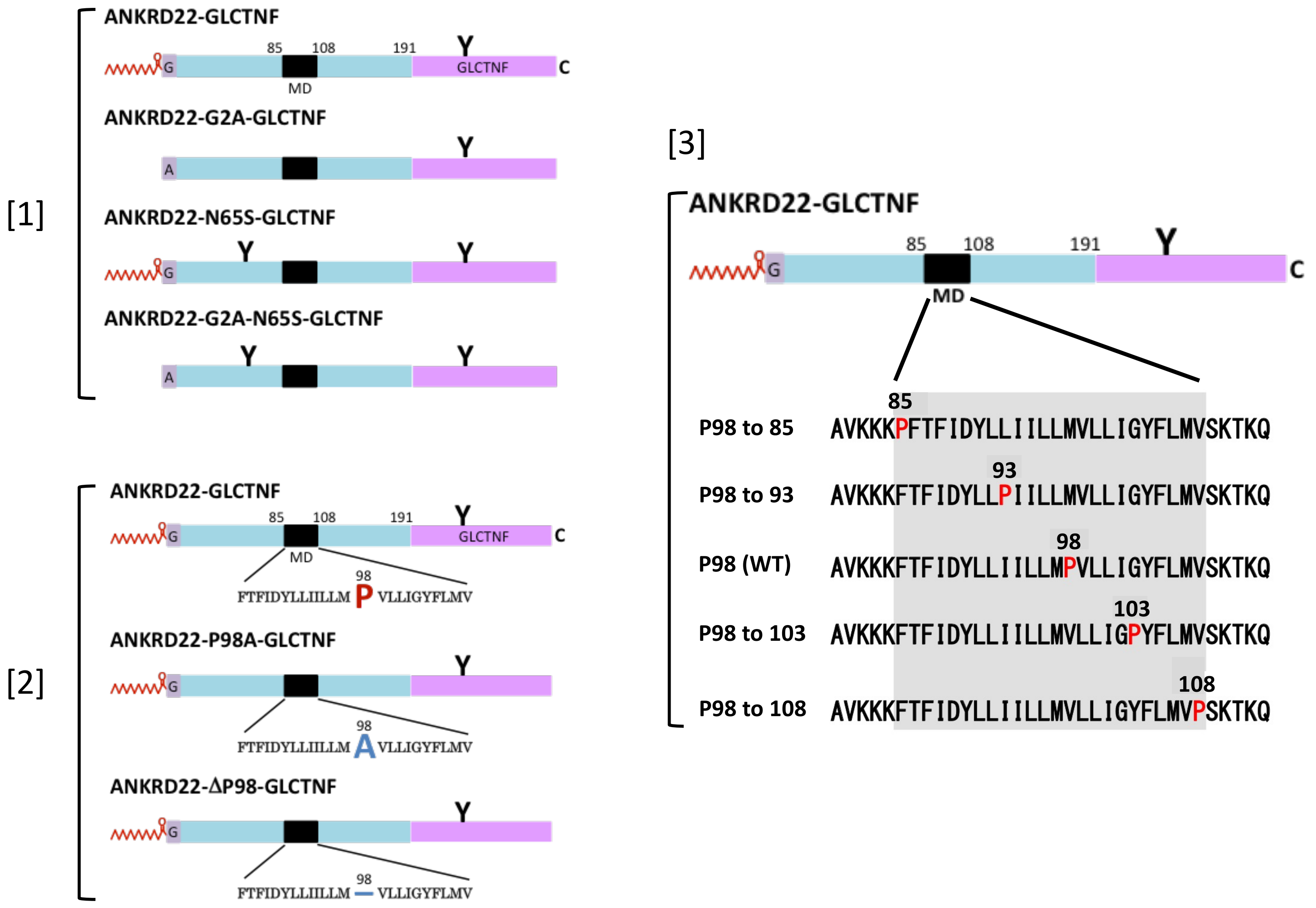

Supplementary Figure S5

The name and structure of ANKRD22-GLCTNF mutants analyzed in Figure 5.

[1] ANKRD22-GLCTNF mutants analyzed in B and E.
[2] ANKRD22-GLCTNF mutants analyzed in D.
[3] ANKRD22-GLCTNF mutants analyzed in F.

Fig. S5.

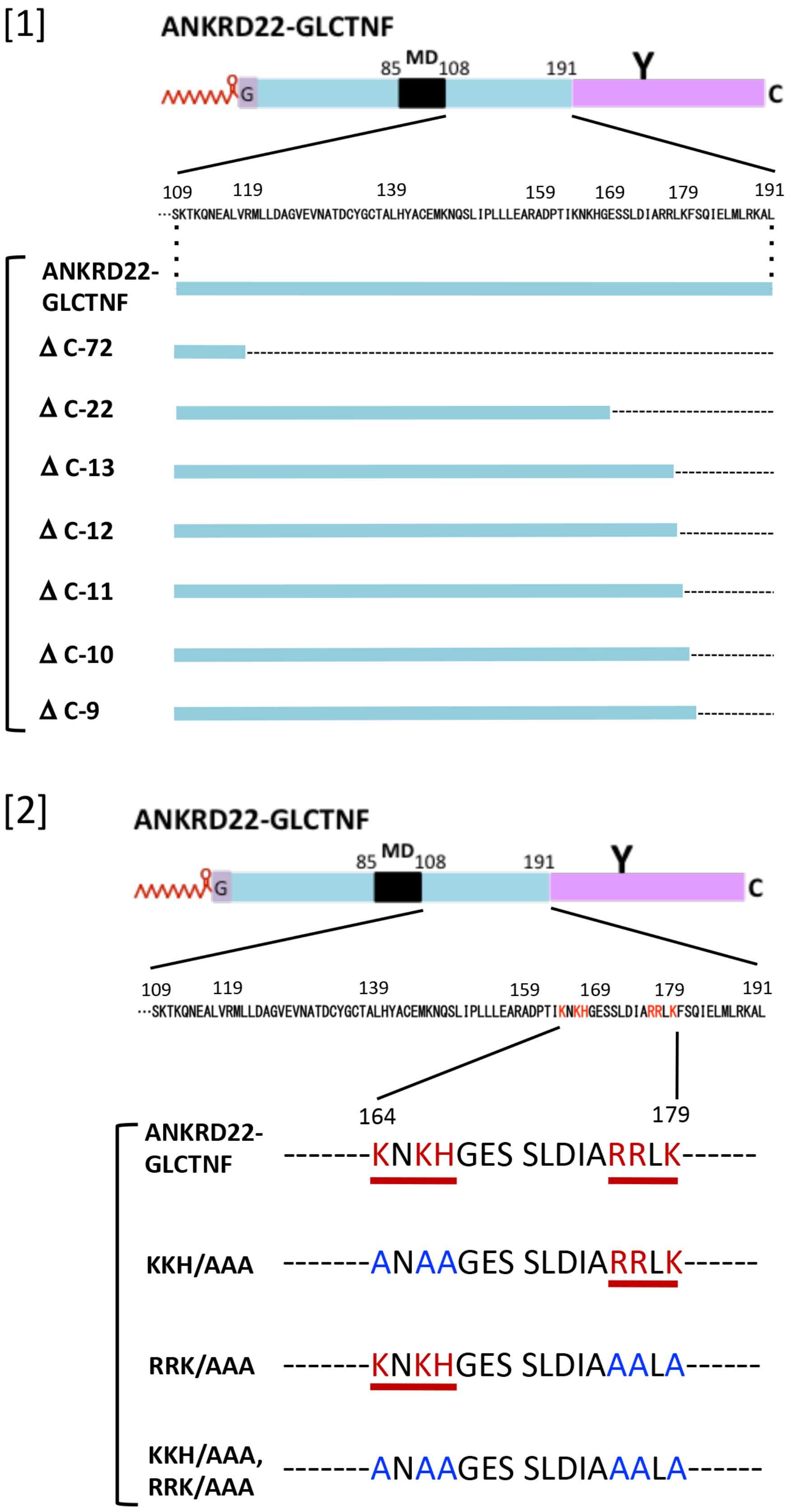

Supplementary Figure S6

The name and structure of ANKRD22-GLCTNF mutants analyzed in Figure 7.
[1] ANKRD22-GLCTNF mutants analyzed in A.
[2] ANKRD22-GLCTNF mutants analyzed in B.

Fig. S6.

Fig 1 [B]

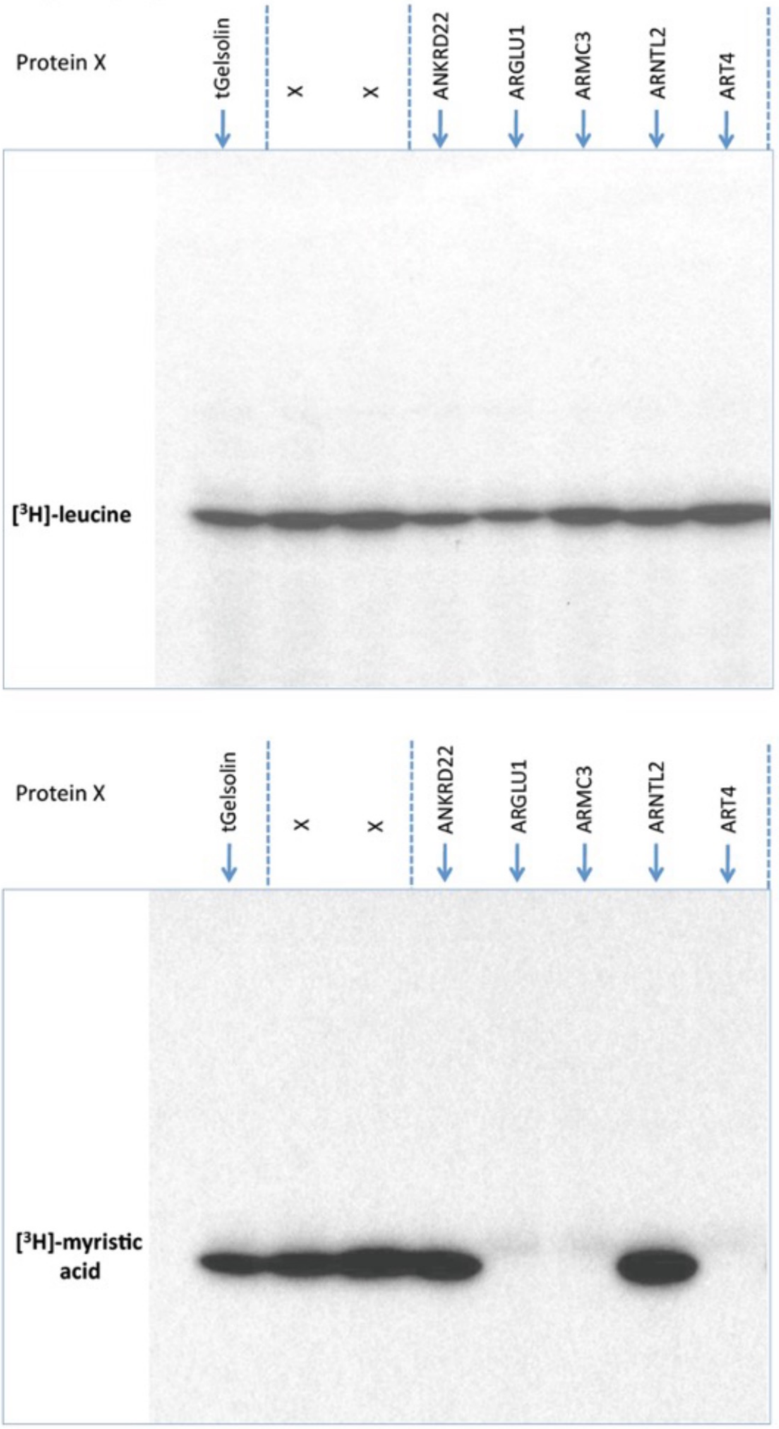

Fig 1 [D]

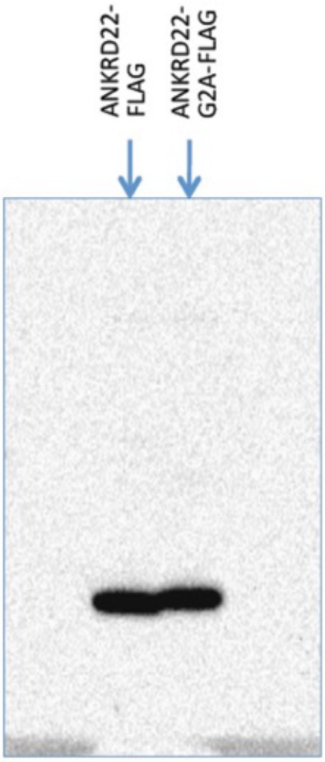

Supplementary Figure S7

Raw images for Fig. 1 B and D.
Lanes marked X have been cropped from the blot shown in Fig. 1B.

Fig. S7.

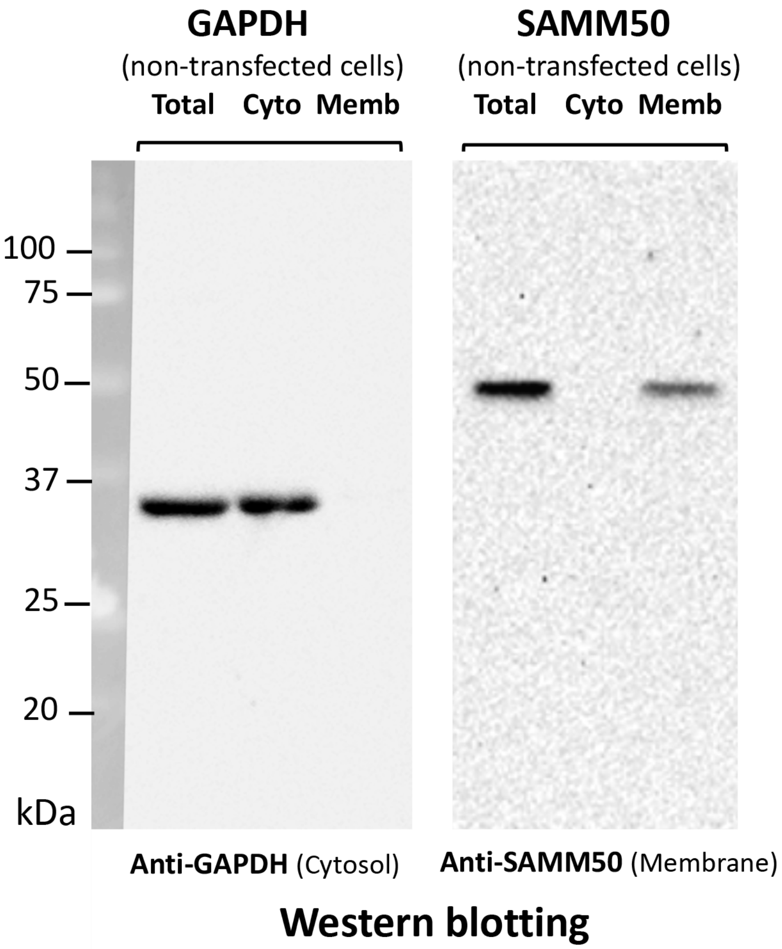

Supplementary Figure S8

Membrane proteins and cytosolic proteins were efficiently fractionated by the membrane extraction kit used in Fig. 4 A.

When the total cell lysates of non-transfected COS-1 cells were fractionated into cytosolic and membrane fraction using the membrane extraction kit, endogenous SAMM50, a membrane protein marker, was detected exclusively in the membrane fraction, whereas GAPDH, a cytosolic marker protein, was detected exclusively in the cytosolic fraction.

Fig. S8.

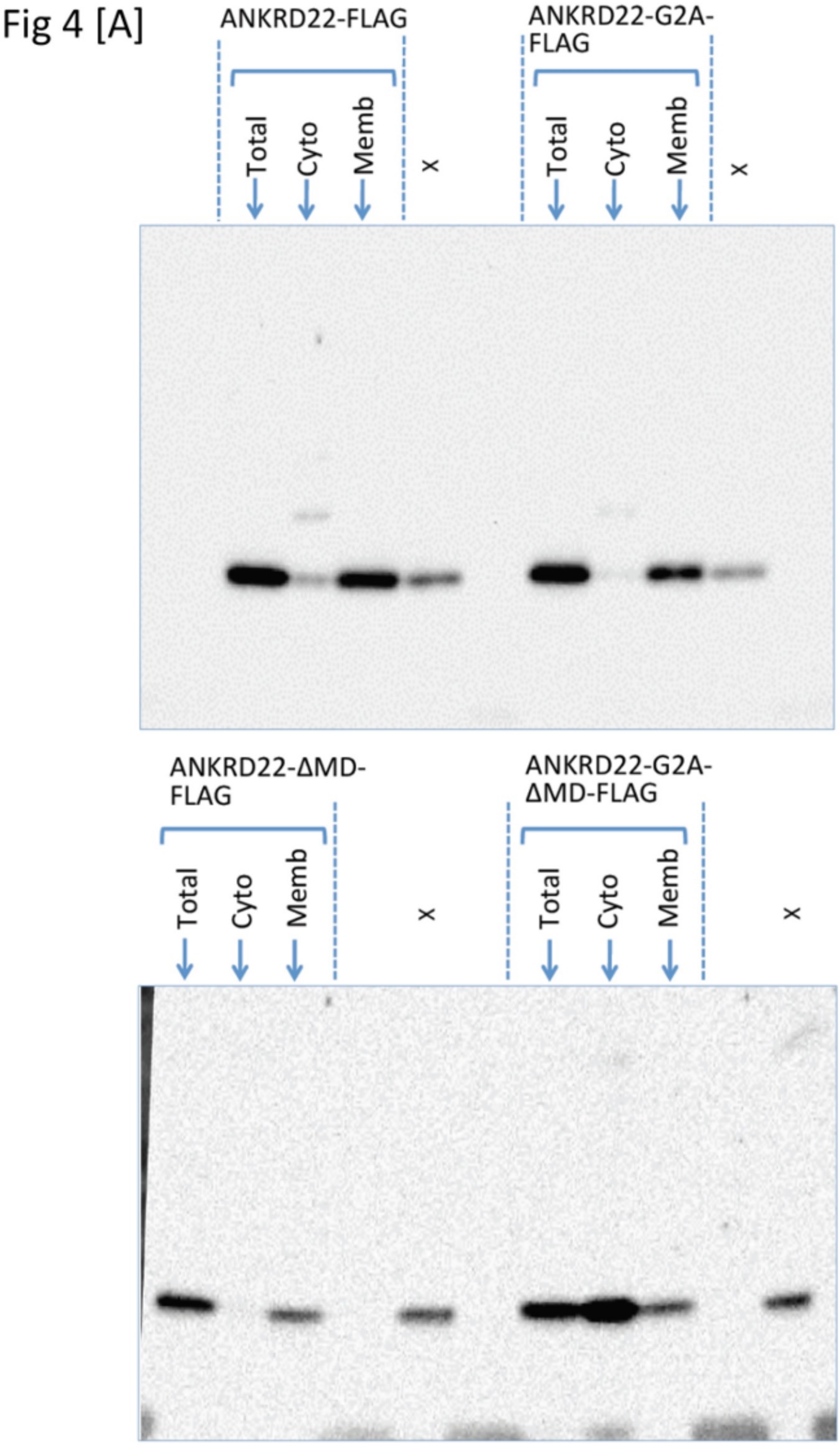

Supplementary Figure S9

Raw images for Fig. 4A.
Lanes marked X have been cropped from the blots shown in Fig. 4A.

Fig. S9.

Fig 5 [A]

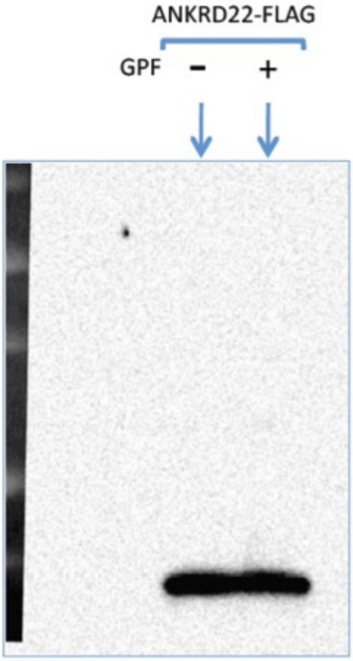

Fig 5 [B]

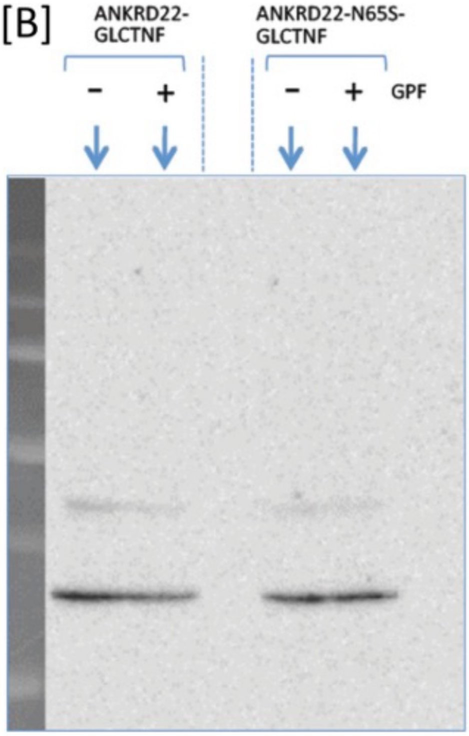

Fig 5 [D]

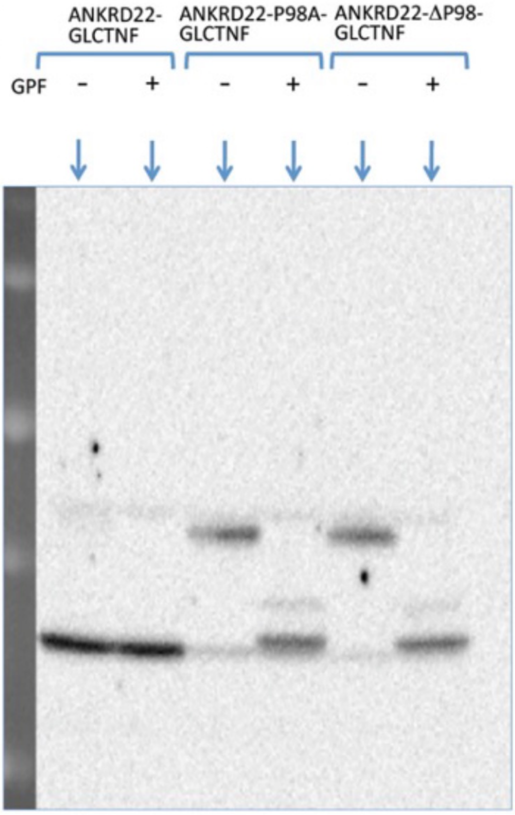

Supplementary Figure S10

Raw images for Fig. 5 A, B, and D.

Fig. S10.

Fig 5 [E]

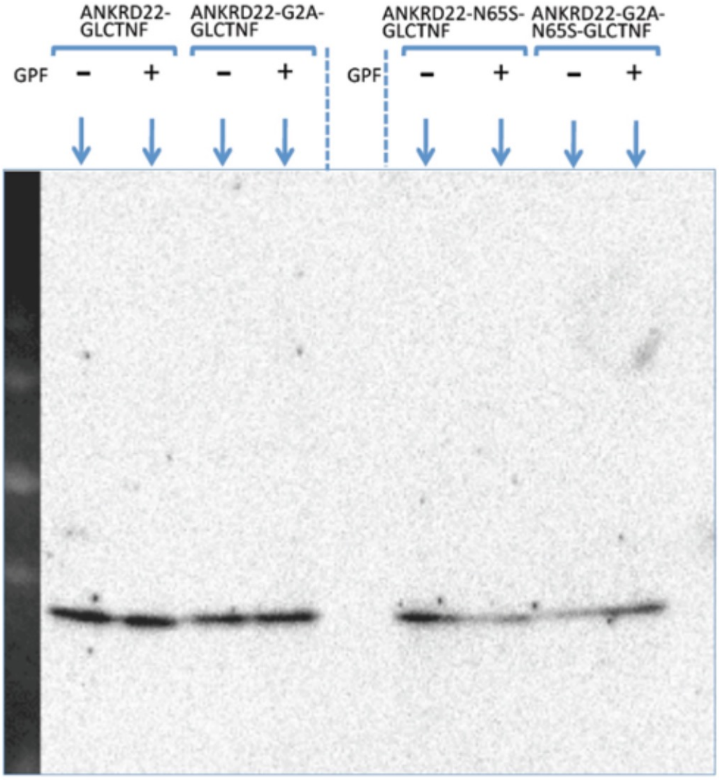

Fig 5 [F]

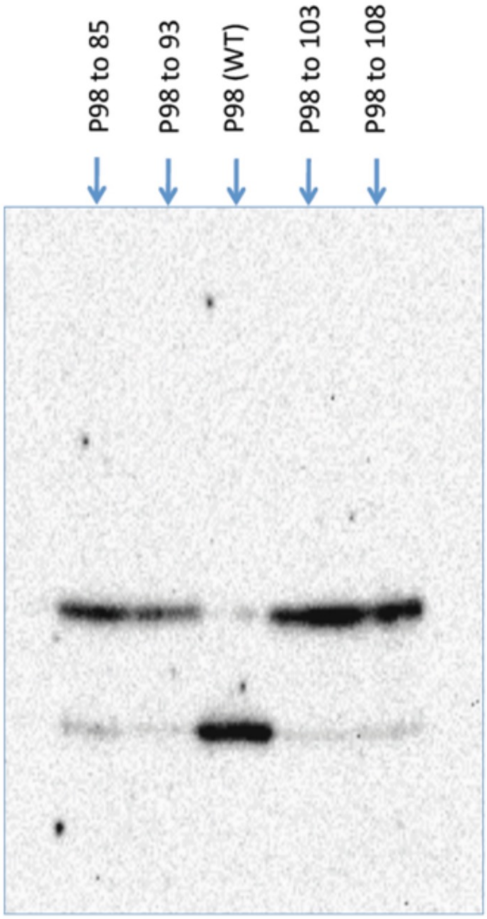

Supplementary Figure S11

Raw images for Fig. 5 E and F.

Fig. S11.

Supplementary Table S1.

The nucleotide sequences of oligonucleotides used to construct pTD1-X(N10)-tGelsolin

Primer	Sequence
ANKRD22N10-N	5’-atcatgggaatcctatactctgagcccatctgcg-3’
ANKRD22N10-C	aattcgcagatgggctcagagtataggattcccatgat
ARGLU1N10-N	atcatgggccggtctcggagccggagctcgtccg
ARGLU1N10-C	aattcggacgagctccggctccgagaccggcccatgat
ARMC3N10-N	atcatgggtaaaaagataaagaaggaagtagagg
ARMC3N10-C	aattcctctacttccttctttatctttttacccatgat
ARNTL2N10-N	atcatggggtctttcagctcacacatgacagagg
ARNTL2N10-C	aattcctctgtcatgtgtgagctgaaagaccccatgat
ART4N10-N5	atcatgggtccattgatcaacagatgcaagaagg
ART4N10-C5	aattccttcttgcatctgttgatcaatggacccatgat

Table. S1.

Supplementary Table S2.
The nucleotide sequences of oligonucleotide primers used for PC

Primer	Sequence
Primer-N1	5’-gcgcgaattcatgggaatcctatac-3’
Primer-C1	gcgcgatatccaatgctttccttag
Primer-N2	gatcggatccatgggaatcctatac
Primer-C2	gcgcgaattccaatgctttccttag
Primer-N3	gatcggatccatgggaatcctatac
Primer-C3	gatcgaattctcgtacaagagcctc
Primer-N4	gatcggatccatgggaatcctatac
Primer-C4	gcgcgaattcctcaccatgcttatt
Primer-N5	gatcggatccatgggaatcctatac
Primer-C5	gcgcgaattctaatctccgtgcaat
Primer-N6	gatcggatccatgggaatcctatac
Primer-C6	gcgcgaattcttttaatctccgtgc
Primer-N7	gatcggatccatgggaatcctatac
Primer-C7	gcgcgaattcaaattttaatctccg
Primer-N8	gatcggatccatgggaatcctatac
Primer-C8	gcgcgaattcaaattttaatctccg
Primer-N9	gatcggatccatgggaatcctatac
Primer-C9	gcgcgaattcctgggaaaattttaa

Table. S2.

Supplementary Table S3.

The nucleotide sequences of oligonucleotides used for ANKRD22 mutagenesis

Primer	Sequence
G2A-F	5’-ttcatggcaatcctatactctgagcc-3’
G2A-R	taggattgccatgaattccagcacac
ΔMD-F	gaaaaaagtatcaaagacaaagcag
ΔMD-R	tttgatactttttcttcacagcata
P98A-F	ttaatggctcttctgcttattgggtat
P98A-R	ttaatggctcttctgcttattgggtat
ΔP98-F	cttaatggttctgcttattgggtat
ΔP98-R	agcagaaccattaagaggataattag
P85-F	gaaaaaaccttttaccttcattgattat
P85-R	aggtaaaaggtttttcttcacagcata
P93-F	ctactacctattatcctcttaatggtt
P93-R	gataataggtagtagataatcaatgaa
P103-F	attgggccttatttcctcatggtatca
P103-R	attgggccttatttcctcatggtatca
P108-F	atggtaccttcaaagacaaagcagaat
P108-R	ctttgaaggtaccatgaggaaataccc
G2A'-F	tccatggcaatcctatactctgagccc
G2A'-R	taggattgccatggatccgagctcggt
KKH/AAA-F	tagcgaatgcggctggtgagagctcactggat
KKH/AAA-R	cagccgcattcgctattgtggggtctgcacg
RRK/AAA-F	cagcggcattagcattttcccagattgaatta
RRK/AAA-R	atgctaatgccgctgcaatatccagtgagct
N65S-F	aatgctagtgtcaacctcaaaaaccag
N65S-R	gaagacactagcatttcttcttaaaag
ΔFlag-F	agcattgtaagcggccgctcgagca
ΔFlag-R	gccgcttacaatgctttccttagcat

Table. S3.

Supplementary Table S4.

The strategies for construction of pcDNA3 plasmids including cDNA coding ANKRD22 mutants by PCR

	Name of plasmid	Strategy for plasmid construction
1	pcDNA3-ANKRD22-FLAG	product of PCR with pUC57-ANKRD22 as the template and primers Primer-N1 and Primer-C1 was BamHI/EcoRI cloned into pcDNA3-FLAG
2	pcDNA3-ANKRD22-GLCTNF	product of PCR with pcDNA3-ANKRD22-FLAG as the template and primers Primer-N2 and Primer-C2 was BamHI/EcoRI cloned into pcDNA3-GLCTNF
3	pcDNA3-ANKRD22-ΔC-72-GLCTNF	product of PCR with pcDNA3-ANKRD22-FLAG as the template and primers Primer-N3 and Primer-C3 was BamHI/EcoRI cloned into pcDNA3-GLCTNF
4	pcDNA3-ANKRD22-ΔC-22-GLCTNF	product of PCR with pcDNA3-ANKRD22-FLAG as the template and primers Primer-N4 and Primer-C4 was BamHI/EcoRI cloned into pcDNA3-GLCTNF
5	pcDNA3-ANKRD22-ΔC-13-GLCTNF	product of PCR with pcDNA3-ANKRD22-GLCTNF as the template and primers Primer-N5 and Primer-C5 was BamHI/EcoRI cloned into pcDNA3-GLCTNF
6	pcDNA3-ANKRD22-ΔC-12-GLCTNF	product of PCR with pcDNA3-ANKRD22-GLCTNF as the template and primers Primer-N6 and Primer-C6 was BamHI/EcoRI cloned into pcDNA3-GLCTNF
7	pcDNA3-ANKRD22-ΔC-11-GLCTNF	product of PCR with pcDNA3-ANKRD22-GLCTNF as the template and primers Primer-N7 and Primer-C7 was BamHI/EcoRI cloned into pcDNA3-GLCTNF
8	pcDNA3-ANKRD22-ΔC-10-GLCTNF	product of PCR with pcDNA3-ANKRD22-GLCTNF as the template and primers Primer-N8 and Primer-C8 was BamHI/EcoRI cloned into pcDNA3-GLCTNF
9	pcDNA3-ANKRD22-ΔC-9-GLCTNF	product of PCR with pcDNA3-ANKRD22-GLCTNF as the template and primers Primer-N9 and Primer-C9 was BamHI/EcoRI cloned into pcDNA3-GLCTNF

Table. S4.

Supplementary Table S5.

The strategies for construction of pcDNA3 plasmids including cDNA coding ANKRD22 mutants by site-directed mutagenesis

	Name of plasmid	Strategy for plasmid construction
1	pcDNA3-ANKRD22-G2A-FLAG	Site-directed mutagenesis using mutagenic primers G2A-F and G2A-R with pcDNA3-ANKRD22-FLAG as the template.
2	pcDNA3-ANKRD22-ΔMD-FLAG	Site-directed mutagenesis using mutagenic primers ΔMD-F and ΔMD-R with pcDNA3-ANKRD22-FLAG as the template.
3	pcDNA3-ANKRD22-G2A-ΔMD-FLAG	Site-directed mutagenesis using mutagenic primers ΔMD-F and ΔMD-R with pcDNA3-ANKRD22-G2A-FLAG as the template.
4	pcDNA3-ANKRD22-P98A-GLCTNF	Site-directed mutagenesis using mutagenic primers P98A-F and P98A-R with pcDNA3-ANKRD22-GLCTNF as the template.
5	pcDNA3-ANKRD22-ΔP98-GLCTNF	Site-directed mutagenesis using mutagenic primers ΔP98-F and ΔP98-R with pcDNA3-ANKRD22-GLCTNF as the template.
6	pcDNA3-ANKRD22-P98 to 85-GLCTNF	Site-directed mutagenesis using mutagenic primers P85-F and P85-R with pcDNA3-ANKRD22-ΔP98-GLCTNF as the template.
7	pcDNA3-ANKRD22-P98 to 93-GLCTNF	Site-directed mutagenesis using mutagenic primers P93-F and P93-R with pcDNA3-ANKRD22-ΔP98-GLCTNF as the template.
8	pcDNA3-ANKRD22-P98 to 103-GLCTNF	Site-directed mutagenesis using mutagenic primers P103-F and P103-R with pcDNA3-ANKRD22-ΔP98-GLCTNF as the template.
9	pcDNA3-ANKRD22-P98 to 108-GLCTNF	Site-directed mutagenesis using mutagenic primers P108-F and P108-R with pcDNA3-ANKRD22-ΔP98-GLCTNF as the template.
10	pcDNA3-ANKRD22-G2A-GLCTNF	Site-directed mutagenesis using mutagenic primers G2A'-F and G2A'-R with pcDNA3-ANKRD22-GLCTNF as the template.
11	pcDNA3-ANKRD22-KKH/AAA-GLCTNF	Site-directed mutagenesis using mutagenic primers KKH/AAA-F and KKH/AAA-R with pcDNA3-ANKRD22-GLCTNF as the template.
12	pcDNA3-ANKRD22-RRK/AAA-GLCTNF	Site-directed mutagenesis using mutagenic primers RRK/AAA-F and RRK/AAA-R with pcDNA3-ANKRD23-GLCTNF as the template.
13	pcDNA3-ANKRD22-KKH/AAA, RRK/AAA-GLCTNF	Site-directed mutagenesis using mutagenic primers RRK/AAA-F and RRK/AAA-R with pcDNA3-ANKRD22-KKH/AAA-GLCTNF as the template.
14	pcDNA3-ANKRD22-N65S-GLCTNF	Site-directed mutagenesis using mutagenic primers N65S-F and N65S-R with pcDNA3-ANKRD22-GLCTNF as the template.
15	pcDNA3-ANKRD22-G2A-N65S-GLCTNF	Site-directed mutagenesis using mutagenic primers N65S-F and N65S-R with pcDNA3-ANKRD22-G2A-GLCTNF as the template.
16	pcDNA3-ANKRD22-no tag	Site-directed mutagenesis using mutagenic primers ΔFlag-F and ΔFlag-R with pcDNA3-ANKRD22-FLAG as the template.

Table. S5.

Supplementary Table S6.
The concentration of fluorescent probes and 1st- and 2nd-antibodies used in the immunofluorescence analysis.

Figure No.	1st antibody		2nd antibody		Fluoresecent dye 1		Fluoresecent dye 2	
Fig.2. top	anti-FLAG	4 µg/ml	anti-Mouse IgG-FITC	7 µg/ml	Hoechst	1 µg/ml	MitoTracker	1 µM
Fig.2. middle	anti-FLAG	4 µg/ml	anti-Mouse IgG-ALEXA 594	4 µg/ml	Hoechst	1 µg/ml		
Fig.2. bottom	anti-FLAG	4 µg/ml	anti-Mouse IgG-ALEXA 594	4 µg/ml	Hoechst	1 µg/ml	Lipi-Green	0.1 µM
Fig.3.	anti-FLAG	4 µg/ml	anti-Mouse IgG-ALEXA 594	4 µg/ml	Hoechst	1 µg/ml	Lipi-Green	0.1 µM
Fig.4.	anti-FLAG	4 µg/ml	anti-Mouse IgG-ALEXA 594	4 µg/ml	Hoechst	1 µg/ml	Lipi-Green	0.1 µM
Fig.6.	anti-TNF	1.3 µg/ml	anti-Mouse IgG-ALEXA 594	4 µg/ml	Hoechst	1 µg/ml	Lipi-Green	0.1 µM
Fig.7.	anti-TNF	1.3 µg/ml	anti-Mouse IgG-ALEXA 594	4 µg/ml	Hoechst	1 µg/ml	Lipi-Green	0.1 µM
Fig.8.	anti-TNF	1.3 µg/ml	anti-Mouse IgG-ALEXA 594	4 µg/ml	Hoechst	1 µg/ml	Lipi-Green	0.1 µM
Suppl. Fig.S1. upper	anti-FLAG	1.2 µg/ml	anti-Mouse IgG-ALEXA 594	4 µg/ml	Hoechst	1 µg/ml	Lipi-Green	0.1 µM
Suppl. Fig.S1. lower	anti-FLAG	1.2 µg/ml	anti-Mouse IgG-FITC	7 µg/ml	Hoechst	1 µg/ml	Nile Red	0.2 µg/ml
Suppl. Fig.S2.	anti-FLAG	1.2 µg/ml	anti-Mouse IgG-ALEXA 594	4 µg/ml	Hoechst	1 µg/ml	Lipi-Green	0.1 µM
Suppl. Fig.S3.	anti-ANKRD22	1 µg/ml	anti-Mouse IgG-ALEXA 594	4 µg/ml	Hoechs	1 µg/ml	Lipi-Green	0.1 µM
Suppl. Fig.S4.	anti-FLAG	1.2 µg/ml	anti-Mouse IgG-ALEXA 594	4 µg/ml	Hoechst	1 µg/ml	Lipi-Green	0.1 µM

Table. S6.
